# Progress of personalized medicine of cystic fibrosis in the times of efficient CFTR modulators

**DOI:** 10.1186/s40348-025-00194-0

**Published:** 2025-05-05

**Authors:** Burkhard Tümmler, Sophia Theres Pallenberg, Anna-Maria Dittrich, Simon Y. Graeber, Lutz Naehrlich, Olaf Sommerburg, Marcus A. Mall

**Affiliations:** 1 Department of Pediatric Pneumology, Allergology and Neonatology, Hannover Medical School, Carl-Neuberg-Str. 1, Hannover, 30625 Germany; 2 German Center for Lung Research, Biomedical Research in Endstage and Obstructive Lung Disease (BREATH), Hannover Medical School, Hannover, Germany; 3 Department of Pediatric Respiratory Medicine, Immunology and Critical Care Medicine, and Cystic Fibrosis Center, Charité - Universitätsmedizin Berlin, Corporate Member of Freie Universität Berlin and Humboldt-Universität Zu Berlin, Berlin, Germany; 4 Berlin Institute of Health (BIH) at Charité - Universitätsmedizin Berlin, Berlin, Germany; 5 German Center for Lung Research (DZL), Associated Partner Site, Berlin, Germany; 6 Department of Pediatrics, Justus Liebig University Giessen, Giessen, Germany; 7 Universities of Giessen and Marburg Lung Center (UGMLC), German Center for Lung Research (DZL), Giessen, Germany; 8 Division of Pediatric Pneumology and Allergy, and Cystic Fibrosis Center, Department of Pediatrics, University of Heidelberg, Heidelberg, Germany; 9 Translational Lung Research Center Heidelberg (TLRC), German Center for Lung Research (DZL),, University of Heidelberg, Heidelberg, Germany

**Keywords:** CFTR, Biomarker, Cystic fibrosis, Elexacaftor, Gene therapy, Ivacaftor, Tezacaftor, Theratyping

## Abstract

**Background:**

Cystic fibrosis (CF) is a systemic disorder of exocrine glands that is caused by mutations in the CFTR gene.

**Main body:**

The basic defect in people with CF (pwCF) leads to impaired epithelial transport of chloride and bicarbonate that can be assessed by CFTR biomarkers, i.e. the β-adrenergic sweat rate and sweat chloride concentration (SCC), chloride conductance of the nasal respiratory epithelium (NPD), urine secretion of bicarbonate, intestinal current measurements (ICM) of chloride secretory responses in rectal biopsies and in bioassays of chloride transport in organoids or cell cultures. CFTR modulators are a novel class of drugs that improve defective posttranslational processing, trafficking and function of mutant CFTR. By April 2025, triple combination therapy with the CFTR potentiator ivacaftor (IVA) and the CFTR correctors elexacaftor (ELX) and tezacaftor (TEZ) has been approved in Europe for the treatment of all pwCF who do not carry two minimal function CFTR mutations. Previous phase 3 and post-approval phase 4 studies in pwCF who harbour one or two alleles of the major mutation F508del consistently reported significant improvements of lung function and anthropometry upon initiation of ELX/TEZ/IVA compared to baseline. Normalization of SCC, NPD and ICM correlated with clinical outcomes on the population level, but the restoration of CFTR function was diverse and not predictive for clinical outcome in the individual patient. Theratyping of non-F508del CF genotypes in patient-derived organoids and cell cultures revealed for most cases clinically meaningful increases of CFTR activity upon exposure to ELX/TEZ/IVA. Likewise, every second CF patient with non-F508del genotypes improved in SCC and clinical outcome upon exposure to ELX/TEZ/IVA indicating that triple CFTR modulator therapy is potentially beneficial for all pwCF who do not carry two minimal function CFTR mutations. This group who is not eligible for CFTR modulators may opt for gene addition therapy in the future, as the first-in-human trial with a recombinant lentiviral vector is underway.

**Future directions:**

The upcoming generation of pwCF will probably experience a rather normal life in childhood and adolescence. To classify the upcoming personal signatures of CF disease in the times of efficient modulators, we need more sensitive CFTR biomarkers that address the long-term course of airway and gut microbiome, host defense, epithelial homeostasis and multiorgan metabolism.

## Background

### Prologue: The Osler – Garrod contrast

Barton Childs (1916–2010) was an American pediatrician and geneticist, who defined the field of genetic medicine and provided the best rationale for its existence. In his 1999 book, Genetic Medicine: A Logic of Disease [1], he introduced his concept of disease as disturbed homeostasis due to interacting genetic and societal factors. Taking the contrast between William Osler (1849–1919) and Archibald Garrod (1857–1936), two of the most influential physicians in the early twentieth century, Childs argued that in the future, all medicine, or medical theory, must be based on the individuality of gene – environment interaction. Osler, the activist, conjured with facts; Garrod, a contemplative man, with ideas. The Oslerian spirit of medicine shaping our todays consensus guidelines (’Leitlinien’) reflects the emphasis on the disease and how its effects are to be reversed. The patient, who is perceived as representative of the class of people with the disease at hand, might be anybody. In other words, Oslerian thinking is organized about treatment and management. In contrast, Garrod saw the patient as a less well adapted product of evolution and the disease as a consequence of a unique individual’s encounter with an environment for which he was uniquely unfit. Susceptibility to disease is a consequence of the chemical and biological individuality among patients. Thus, Garrodian thinking paved the way to our present concept of personalized medicine. Childs concluded: ‘No one would deny that Osler was the hero of the medicine of the twentieth century. It is likely that Garrod will be the icon of the twenty-first’ [1].

Here, writing about people with cystic fibrosis (pwCF) will be inspired by Garrodian thinking decorated by Oslerian notes if necessary.

### In brief: Cystic fibrosis – status 2025

Cystic fibrosis (CF) is a systemic disorder of exocrine glands that primarily affects the respiratory, gastrointestinal, hepatobiliary and reproductive tracts [2, 3]. CF is an autosomal recessive disease affecting more than 190,000 people worldwide [4] that is caused by two mutations *in trans* in the *Cystic Fibrosis Transmembrane Conductance Regulator* (*CFTR*) gene [5]. The universal feature of CF organ damage caused by CFTR deficiency is the buildup of mucus that is thick, tenacious, and dehydrated [6]. Thanks to continuously improved symptomatic treatment during the last five decades [7–9] this lethal pediatric disease has been transformed into a chronic disorder with a median life expectancy of nowadays more than 60 years [7, 10].

During the last decade CFTR modulators have arrived at the clinic [11] that target the basic defect in CF of impaired epithelial conductance for chloride and bicarbonate [12–14]. There are two classes of CFTR modulators: Potentiators [15–17] increase the activity of CFTR to the cell surface and correctors facilitate the translation, folding, maturation and trafficking of mutant CFTR to the cell surface and/or prevent its premature degradation [18, 19]. By 2019 a highly effective triple combination of the potentiator ivacaftor (IVA) [15] and the two correctors elexacaftor (ELX) [20] and tezacaftor (TEZ) [21, 22] have become available for the treatment of the more than 80% of pwCF who harbor at least one *CFTR* allele that is responsive to this medication [23–25]. ELX/TEZ/IVA is the first CFTR modulator therapy shown to halt lung function decline over an extended time period [26, 27]. Based on an individual person-level microsimulation model the median lifetime survival of pwCF receiving triple modulator therapy and best supportive care has been estimated to be 72 years [28].

Although CF is a rare disease, its advances in diagnosis, therapy and management have it made a showcase for the potential achievements of modern medicine reflected by more than 65,000 entries in the PubMed database by the end of 2024.

## Main text

### Cystic fibrosis – a monogenic disease personalized by hundreds of disease-causing mutations in the *CFTR* gene

CF is caused by two mutations *in trans* in the *CFTR* gene [5]. As of the most recent file (25 September 2024), 1,167 sequence variants are annotated on the CFTR2 website, 1,085 of which were classified as disease-causing [29]. By December 2024, CFTR France had classified 989 sequence variants, about 80% of which are nucleotide substitutions and the remaining 20% are insertions, deletions, repeats or rearrangements of the *CFTR* sequence [30]. The DNA alterations lead either to amino acid substitutions (67.7%) or to premature stop codons (14.8%), frameshifts of the reading frame (12.6%), gain or loss of amino acids (1.3%) or to no change of the reading frame (3.6%). Of the 989 sequence variants, 500 and 76 variants were discerned as disease-causing and non disease-causing (‘benign polymorphisms’), respectively [31]. By combining in silico predictions and wet lab, clinical and epidemiology data, the remaining sequence variants were assigned to the intermediate categories of ‘likely benign’ (0.7%), ‘likely pathogenic’ (4.6%) or variants of unknown significance (VUS, 36.5%) [31]. VUS carriers may be clinically asymptomatic or may present the features of mild CF or CFTR-related disorder (CFTR-RD) [32, 33] such as congenital absence of the vas deferens (CAVD), diffuse bronchiectasis, chronic rhinosinusitis, chronic or acute recurrent pancreatitis, allergic bronchopulmonary aspergillosis (ABPA), primary sclerosing cholangitis and aquagenic wrinkling [34, 35]. These eight conditions typically have an etiology unrelated to CFTR. Since *CFTR* genetics is not informative, any suspect case of CFTR-RD has to be examined by CFTR biomarkers (see below) to make a diagnosis [33].

The most common disease-causing *CFTR* mutation, the 3-bp in frame deletion F508del (c.1521_1523 delCTT; p.Phe508 del according to the HGVS Nomenclature [36]) accounts worldwide for approximately 60—70% of all CF chromosomes with variable frequencies depending on populations and geographical locations [5]. Within Europe, the frequency varies from 32% in Turkey to 83% in Denmark [5, 37]. The majority of other mutations is rare. For example, in Germany just eight disease-causing *CFTR* mutations are present at a frequency of more than 1% in the population of CF alleles [10].

### Molecular pathology of CFTR mutations

The ABC transporter CFTR (ABCC7) is a low-conductance anion-selective ion channel with complex regulation [38–40], namely CFTR activation by protein kinase dependent phosphorylation and CFTR channel gating by ATP binding and hydrolysis [39–41]. CFTR is assembled from 1,480 amino acids organized into five major domains, i.e. two membrane-spanning domains (MSDs), two nucleotide-binding domains (NBDs) and a regulatory domain (RD) arranged in two MSD-NBD motifs separated by the RD [38, 42, 43]. The MSDs are composed of six transmembrane segments (TMs), which assemble to form a gated pore for transmembrane anion flow [44, 45]. The pore is accessible from the cytosol through a large inner vestibule and opens to the extracellular solvent through a narrow portal made up by the extracellular ends of TMs 1, 6, and 8 [46].

CFTR transports chloride to control fluid absorption or secretion by epithelia and conducts bicarbonate to regulate intra- and extracellular pH [12–14]. Depending on the amount of residual CFTR activity conferred by the *CFTR* mutation genotype, CFTR function may be absent, modified and/or reduced in pwCF. Based on functional criteria related to chloride transport, *CFTR* mutations have been assigned to six classes [47]:

- Class I (minimal function mutations) includes structural variants such as large deletions or rearrangements and sequence variants that result in a premature termination codon (PTC) or cause a shift in the reading frame.
- Class II mutations lead to defective posttranslational maturation and/or trafficking so that no or only small amounts of mutant CFTR are translocated to the apical membrane of epithelial cells.
- Class III mutants are defective in the regulation and gating of the ion channel.
- Class IV mutants have altered channel properties (open probability, open time, ion selectivity, conductance).
- Class V mutants are characterized by a reduced amount of normal CFTR protein.
- Class VI mutants have a reduced half-life leading to the absence or severe reduction of CFTR at the membrane surface.

Patients who harbor two class 1, 2 or 3 *CFTR* alleles typically suffer from the whole spectrum of CF disease including exocrine pancreatic insufficiency (PI) [48]. Individuals with two *CFTR* mutations *in trans* who carry at least one class 4 or 5 *CFTR* allele are affected by milder forms of CFTR-associated disease; i.e. CFTR-RD or CF with exocrine pancreatic sufficiency (PS) [48].

### Diversity of patients’ basic defect assessed by CFTR biomarkers

The *CFTR* mutation genotype translates into defects that disrupt CFTR production, channel activity and/or regulatory function. CF is a systemic disorder of exocrine epithelia. However, in CF patients the investigation of the basic defect is currently limited to few organs and cell types, namely the sweat gland, the respiratory, renal and intestinal epithelium and host defense cells [33]. The repertoire may be increased in the next years by protocols that differentiate CF patient-derived induced pluripotent stem cell lines into cell types of interest [49]. Here we describe the currently available CFTR biomarkers sorted by organ.

#### Sweat gland: CFTR-mediated secretion in the coil and chloride reabsorption from the duct

The sweat gland provides two biomarkers of CFTR activity: a linear readout via the ß-adrenergic sweat rate [50] and a logarithmic readout via sweat chloride concentration [51]. Sato and Sato [52] as well as Behm et al. [53] discovered in the 1980 s that CFTR-mediated sweat secretion in the secretory coil can be stimulated with ß-adrenergic agonists if the thermoregulative cholinergic sweat secretion is simultaneously inhibited with atropine. Current ß-adrenergic sweat secretion assays measure the sweat gland secretory responses to this stimulation sequence by either evaporimetry [54, 55] or by recording the growth of single droplets under a microscope [50, 55–57]. Protocols have recently been developed that determine sweat secretion rates by automatic recording, processing and quality control of the kinetics of sweat droplet formation [56, 57]. The ß-adrenergic sweat rate increases approximately linear with CFTR function. The sweat rate is close to 50% in heterozygous carriers of a *CFTR* mutation (median: 0.26 nl/min) compared to healthy controls (median: 0.44 nl/min) and is absent or barely detectable in pwCF (median: 0.006 nl/min) [57]. Thus, the ß-adrenergic sweat secretory test is the first CFTR biomarker assay indicating half-maximal CFTR function in heterozygous gene carriers. It also identifies all pwCF including those carrying mutations associated with normal or borderline sweat chloride concentrations in the quantitative pilocarpine iontophoresis sweat test (QPIT) [58].

QPIT [58, 59] assesses the CFTR-mediated reabsorption of chloride from the primary plasma isotonic sweat in the sweat duct. Based on the abnormally low chloride permeability of the CF sweat duct [60, 61], QPIT is the standard CFTR biomarker in making or excluding a diagnosis of CF. After cholinergic sweat stimulation with pilocarpine iontophoresis, sweat is collected and its chloride concentration determined. Sweat chloride concentration (SCC) varies between 5 and 140 mmol/L [62], whereby 60 mmol/l is the cutoff for a CF diagnosis [63]. CFTR activity decreases with the logarithm of SCC [51].

Data of the CFF Patient registry collected from 25,753 patients between 1996 and 2009 uncovered an association between the SCC at diagnosis and survival at the population level [64]. Median survival age was significantly different between the three sweat chloride categories (SCC < 60 mmol/L, SCC 60–80 mmol/, SCC > 80 mmol/L), with the < 60 mmol/L group having the highest median survival age, followed by the 60–80 mmol/L group [64]. Likewise, median age of survival was significantly lower for patients with two class I, II or III *CFTR* mutations compared to pwCF carrying at least one class IV or V *CFTR* mutation [64]. Data from twin and sibling studies demonstrated that *CFTR* mutations are the primary determinant of SCC variability (56% of variation) followed by variation over time (age, circadian cycle, day-to-day variation) and environmental factors [62]. The intrapair comparison of SCC of monozygous and dizygous twins suggested that genetic modifiers do not significantly influence the outcome of sweat testing [62]. These findings corroborate the clinical practice that QPIT is the adequate test to diagnose CF.

The SCC of F508del homozygotes in normally distributed around a mean of 101 ± 15 mMol/L [62]. During his 40-year working life, the first author met a few F508del homozygous subjects with a SCC below 70 mmol/L. They all presented at the CF clinic with extremely mild CF disease and a late diagnosis in their 20 s to 70 s in line with the population data that a low SCC is associated with higher survival.

#### CFTR-mediated chloride and bicarbonate homeostasis in airway epithelia and host defense cells

The clinical manifestation of CF is primarily determined by pulmonary, hepatobiliary and intestinal disease that is triggered by the *CFTR* mutation type, but then is predominantly shaped by genetic modifiers and environmental factors [65, 66]. Within the lungs, CFTR regulates the pH and hydration of the airway surface liquid (ASL) [67]. The tasks are distributed to different cell types. The abundant secretory and basal cells secrete chloride [68]. On the other hand, the rare CFTR-rich ionocytes [69, 70] mediate the absorption of chloride and fluid from the ASL [71] and modulate its pH by secreting bicarbonate via CFTR-linked chloride/bicarbonate exchange [72]. These processes can be followed in samples from individual CF patients of any age by taking nasal swabs for immediate omics analysis [73] or set-up of cell cultures [71, 74, 75]. After the initial expansion in co-cultures, the epithelial cells are grown at the air–liquid interface for subsequent biochemical, histological or electrophysiological characterization [75, 76]. The nasal brushings harvest the whole spectrum of ionocytes, basal, club, goblet and ciliated cells so that the patient’s basic defect can be assessed at the level of single cells in a real-world scenario [73]. Moreover, one gets access to the host defense cells [73] including the macrophages that are known to be defective in CFTR-mediated phagosome acidification [77] undermining their intracellular capability to lyse CF pathogens such as *Pseudomonas aeruginosa* [78–80].

Besides these novel CFTR biomarkers at the cellular level, nasal transepithelial potential difference (NPD) measurements represent the CFTR biomarker of the respiratory epithelium at the macroscopic scale [81]. NPD measures the chloride conductance of the nasal respiratory epithelium in vivo. NPD tracings of pwCF are characterized by a more negative basic potential, a higher hyperpolarization potential upon amiloride-induced blockage of sodium conductance and an absent or low depolarization potential upon exposure to chloride-free solution because the CFTR-mediated chloride conductance is low or absent [82–84]. Residual CFTR activity of patients carrying a class IV or V mutation shows up in a small depolarization potential [85, 86]. To differentiate CF from non-CF, NPD scores have been developed that evaluate both the hyper- and the depolarization potential [86, 87].

#### Functional and immunochemical analysis of CFTR in rectal biopsies and organoids

Intestinal current measurements (ICM) of rectal biopsies in Mini-Ussing chambers represent the CFTR biomarker that assesses CFTR-mediated chloride secretion in the colonic epithelium [88–90]. CFTR is the dominant chloride channel in human colon responsible for chloride and fluid secretion. ICM evaluates the chloride secretory responses to cAMP-activation via forskolin/IBMX and cholinergic co-activation via carbachol that are mostly driven by CFTR in healthy non-CF tissue [91, 92]. However, in CF with no or low amounts of functional CFTR the anion secretory activities are substantially mediated by bicarbonate through bestrophin Best2 channels [93], by the Epac-Rap-PLC-[Ca^2+^] signaling pathway [94] and increased by basolateral and apical potassium channels [95, 96]. Thus, to discern CFTR-mediated chloride secretion from other anion secretory activities, one may add at the end of the ICM experiment either the specific CFTR inhibitor CFTR(inh)− 172 [97] or re-stimulate with histamine at a high concentration of DIDS that inhibits all anion channels but CFTR [89, 92]. As in NPD residual CFTR function is typically detected in pwCF who are carrying a class IV or class V mutation on at least one CF allele [98, 99].

The rectal biopsies can be utilized for immunochemical CFTR protein analysis. Immunoblots visualize the CFTR glyco-isoforms. The polypeptide chain is synthesized in the endoplasmic reticulum (ER) (CFTR band A). Its mannose-rich core-glycosylated ER form (CFTR band B) is translocated to the Golgi apparatus for glycan processing before it can reach the apical membrane as complex glycosylated protein (CFTR band C) [100, 101]. The major CF-causing mutation F508del is a class II mutation defective in posttranslational processing and trafficking [101, 102]. Rectal biopsies of F508del homozygous donors contained the F508 del CFTR glycoform C in amounts of 0% to 14% (median: 3%) of that of wild-type CFTR of non-CF donors [103]. Apart from the colon, apical complex-glycosylated F508 del CFTR has also been detected in patients’ airways, gallbladder and intestine, but not in the sweat gland [104, 105].

ICM has to be performed on freshly excised biopsies. Alternatively, the biopsies may be converted to self-renewing and self-organizing organoids that closely resemble the in vivo tissue architecture in terms of cell polarity, self-renewal kinetics and cell-type composition [106, 107]. The organoids contain a central lumen lined by the apical membrane, and multiple crypt domains that harbor the stem cells. A non-CF rectal organoid is filled with fluid and has a cystic, spherical appearance [108]. Raising cAMP concentrations by forskolin results in CFTR channel opening and rapid swelling [49]. Forskolin-induced swelling (FIS) is completely CFTR dependent [49]. CF rectal organoids show strongly reduced or absent forskolin-induced swelling, a lower lumen volume and a more dense and irregular structure [49, 109, 110]. Both the FIS assay and the rectal organoid morphology analysis (ROMA) have become CFTR biomarkers applicable to diagnosis and personalized treatment [49, 110]. In contrast to colonic or rectal organoids, non-CFTR dependent organoid swelling can be observed in organoids derived from airway, kidney, pancreas, bile ducts and epididymis, and swelling can be induced by other agonists [111, 112]. Thus, the easily accessible rectal organoids are particularly suited to assay the CFTR function of individual CF patients.

#### CFTR-dependent renal bicarbonate secretion

The most recently introduced in vivo biomarker of CFTR function is the challenged urine bicarbonate test [113, 114]. The chloride-secreting CFTR channel and the chloride/bicarbonate exchanger pendrin (SLC26A4) are co-localized in the ß intercalated cells of collecting ducts of the kidney [115]. During acute systemic base excess urine secretion of bicarbonate is increased in a CFTR-dependent manner [116], which is examined in the test: First, urine is collected at baseline, and subsequently, each participant ingests 79 mg/kg body weight (corresponding to 0.94 mmol/kg body weight) of NaHCO_3_ dissolved in 200 mL of tap water. Urine is collected hourly for 3 h and 200 mL of tap water is ingested after each urine output for the first 2 h. The functional readout of the test is the total amount of bicarbonate excreted during the 3 h after ingestion of NaHCO_3_ [113, 114]. Bicarbonate secretion was significantly decreased in pwCF with class I or class II mutations, but was not significantly different from healthy controls in pwCF who are harboring a class IV or class V mutation on one CF allele [113, 114].

#### CFTR-mediated interorgan metabolite exchange

Recently, arteriovenous metabolomics in CF newborn pigs have revealed that CFTR is regulating multiorgan metabolism [117]. In CF piglets, the number of metabolites exchanged between the liver and other organs decreased by half compared to that of their wild type littermates (from 140 to 68 metabolites). The number of metabolites transferred to the lung from other organs decreased by fivefold (from 68 to 13 metabolites) whereby particularly the uptake of long chain fatty acids was severely impaired. CFTR loss moreover disrupted the liver release of metabolites and the liver-muscle exchange of glutamate and glutamine. CF kidneys exhibited insufficient reabsorption of many amino acids presumably because the lack of a CFTR anion conductance may change the driving force for sodium and proton-coupled amino acid absorption in the renal proximal tubule. Loss of CFTR also disrupted renal glucose homeostasis because gluconeogenic substrates such as lactate and amino acids were poorly reabsorbed in the proximal tubule. These spectacular, novel findings of the endogenous role of CFTR in interorgan metabolite exchange need to be verified in further work including clinical studies in pwCF until (selected) metabolites will become the next generation of CFTR biomarkers.

### Action of CFTR modulators on mutant CFTR

About 90% of pwCF are homozygous or compound heterozygous for the most common mutation F508del [2–5, 65]. F508del CFTR is defective in posttranslational processing and trafficking [19, 101, 102]. Newly synthesized F508del CFTR fails to adopt a wild-type fold in the endoplasmic reticulum (ER), is targeted to ER-associated degradation and is removed faster from the apical membrane by endocytosis [101, 102]. Consequently, F508del CFTR confers no or low chloride and bicarbonate secretory activity. Correction of the conformational defects of F508del CFTR requires the stabilization of the interfaces between the two NBDs and MSDs (type I corrector) and the stabilization of NBD2 (type II corrector) and F508del NBD1 (type III corrector) [19].

The CFTR modulators lumacaftor (LUM) [18, 118] and tezacaftor (TEZ) [21, 22, 118] are type I correctors [19]. The two drugs stabilize the early steps of F508del CFTR biogenesis at the ER [119, 120], facilitate the subsequent domain assembly in the absence of folded F508del-NBD1 and stabilize the conformation of the folded protein [118–128]. The type III correctors elexacaftor (ELX) [23, 24] and vanzacaftor (VAN) [129] synergistically restore the processing and stabilize the conformation of F508del CFTR in combination with type I or type II correctors [130–134].

The CFTR potentiators ivacaftor (IVA) [15] and deutivacaftor (DVA) [129] enhance channel activity by increasing pore opening while NBDs are dimerized [43]. Channel opening normally requires the binding and subsequent hydrolysis of ATP [40, 41, 135]. Ivacaftor reversibly enhances ATP-independent opening of the channel [136–138] by stabilizing pre-hydrolytic states [139, 140] and thereby improves ion transport in F508del CFTR and overcomes the defective ATP-dependent opening of CF-causing gating mutations [141, 142]. In addition to being a corrector, elexacaftor also act as a co-potentiator of F508del, G551D and M1101K CFTR chloride channels [143–145].

Cryo-electron microscopy of reconstituted recombinant protein revealed that the conformations of wild type CFTR and ELX/TEZ/IVA-bound F508del CFTR were almost indistinguishable from each other indicating that upon binding of the three CFTR modulators F508del CFTR is converted into a wild type conformation [146].

### CFTR biomarker response to triple CFTR modulator therapy with ELX/TEZ/IVA in pwCF harboring one or two F508del alleles

The advent of the triple combination therapy with ELX/TEZ/IVA has been qualified as a game changer for CF [147]. By the end of 2024, nine phase 3 clinical trials [23–25, 148–152] and twenty real-world post-approval studies [26, 27, 153–170] had consistently reported significant improvements of anthropometry (BMI: median 1.3, IQR 1.1—1.6 kg/m^2^) and lung function (ppFEV1: median 11.4, IQR 9.8—13.7%) (Table 1). ELX/TEZ/IVA improved lung ventilation and abnormalities in lung morphology [168, 171–181], including airway mucus plugging, wall thickening and in a few cases even bronchial dilatation [182] and bronchial artery dilatation [183]. Microbial load of the airways with CF pathogens such as *Aspergillus fumigatus* [184, 185]*, Staphylococcus aureus* [186]*, Pseudomonas aeruginosa* [186–188] or nontuberculous mycobacteria [189–191] was reduced, but persisted in most pwCF [186, 192–194] and the microbial network remained vulnerable to fragmentation [195–197]. In the gut, the transit time of food through the small bowel increased [198], but gastrointestinal symptoms remained prevalent [198, 199] or improved only slightly [200–202]. Weight gain was mainly caused by an increased fat mass [203, 204] associated with the risk to develop obesity and metabolic syndrome [204, 205].

**Table 1:**
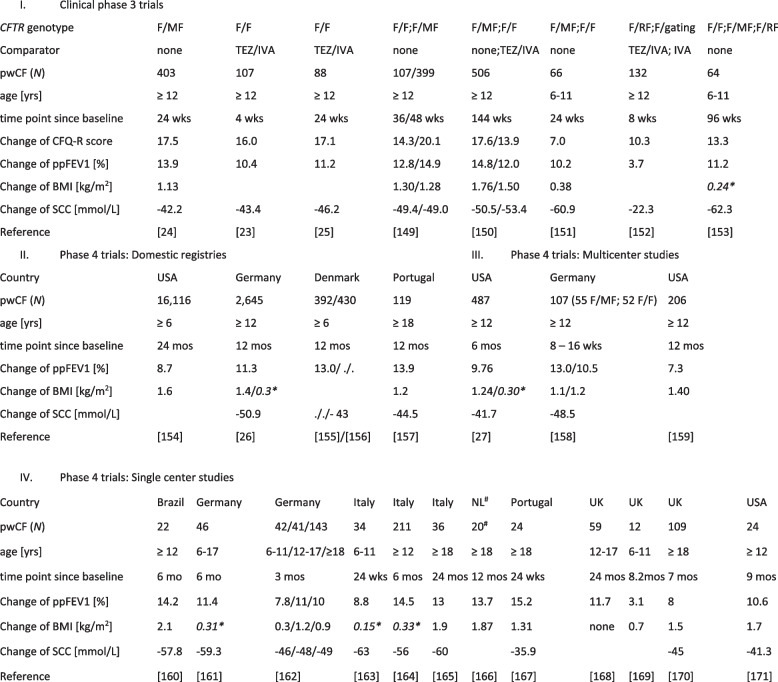
Change of CFQ-R score, ppFEV1, BMI and SCC in pwCF after initiation of triple EXL/TEZ/IVA therapy [23–27, 149–171]

^#^*NL* The Netherlands; *BMI-z-score; *BMI* Body mass index, *CFQ-R* Cystic fibrosis questionnaire – revised application, *F* F508del, *IVA* Ivacaftor, *MF* Minimal function mutation, *ppFEV1* Percentage of predicted forced expiratory volume in 1 s, *pwCF* people with CF, *RF* Residual function mutation, *SCC* Sweat chloride concentration, *TEZ* Tezacaftor

Of the CFTR biomarkers, sweat chloride concentration (SCC) as a surrogate of CFTR activity had commonly been implemented in post-approval studies and as secondary endpoint in the phase 3 trials (Table 1). Upon initiation of therapy with ELX/TEZ/IVA, SCC significantly decreased in adolescents and adults who are homozygous for F508del or compound heterozygous for F508del and a class I mutation. The median value of the median of 21 clinical studies was – 49.0 mmol/L (IQR − 45.0—− 53.4 mmol/L) (Table 1). The decrease of SCC was larger in children [206] and smaller in pwCF who were carrying at least one class IV or class V mutation associated with lower SCC at baseline [151].

SCC shows a log-linear association with CFTR activity [51, 207]. Pooled data of the phase 3 trials with IVA, TEZ/IVA or ELX/TEZ/IVA demonstrated a mean absolute improvement of 7.3%, 16.9%, 19.6% and 20.9% in ppFEV1 in study participants showing a SCC ≥ 80 mmol/L, ≥ 60—≤ 80 mmol/L, > 30—< 60, < 30 mmol/L in sweat test after 24 weeks of modulator therapy, respectively [207]. Likewise, the three groups showed a differential gain of BMI at week 24 of 0.25, 1.02, 1.16 and 1.27 kg/m^2^ [207]. Thus, greater reductions in sweat chloride were associated with improved clinical outcomes. The PROMISE post-approval study [27] confirmed this finding. The change in sweat chloride from baseline to six months after ELX/TEZ/IVA initiation significantly correlated with the improvement in ppFEV1. Linear regression of the SCC data indicated that a decrease of a 10 mM increment in SCC was associated with a mean 0.89 greater change in ppFEV1 [27].

In contrast to the uniform median response of SCC to ELX/TEZ/IVA therapy at the population level, the individual response was heterogeneous [208]. The US CHEC-SC Study group has recently published SCC data from 1,769 pwCF receiving ELX/TEZ/IVA, of whom 56.6% were homozygous for F508del, 24.7% compound heterozygous for F508del and a class I mutation and the remaining 18.7% were carrying other *CFTR* genotypes included in the FDA label [208]. 127 pwCF (7.3%) remained in the classical PI CF range with SCC of more than 80 mmol/L; 243 pwCF (13.7%) had a SCC of 60–80 mmol/L; 939 pwCF (53.1%) had a SSC in the borderline range of 30–60 mmol/L and 460 pwCF (26%) had a SCC in the range of healthy people below 30 mmol/L [208]. Thus, close to 80% of pwCF receiving ELX/TEZ/IVA had crossed the threshold of 60 mmol/l into the non-CF diagnostic range. Conversely, 18% of F508del homozygotes remained in the CF range with SCC ≥ 60 mmol/l compared to 31% of compound heterozygotes for F508 del and a class I mutation. Within subgroups sharing the same *CFTR* genotype, substantial variability of post-ELX/TEZ/IVA SCC was observed in the CHEC-SC study cohort. The US experience was confirmed in CF centers in Europe. For example, the six-month changes from baseline in SCC ranged from − 27.1 to − 76.1 mM for the 10 th to 90 th centile in 211 pwCF seen at the CF center in Milan [163]. In this single center study, the change in SCC weakly correlated with absolute changes of ppFEV1, but did not correlate with the change in BMI [163]. In summary, treatment with ELX/TET/IVA led to a substantial and highly significant decrease of SCC in sweat test at the population level (Table 1), but the response of the individual patient was highly variable. Age, sex, ethnicity and pre-modulator SCC explained less than 10% of the variability in post-ELX/TEZ/IVA SCC in pwCF with the same *CFTR* genotype [208].

**Fig. 1:**
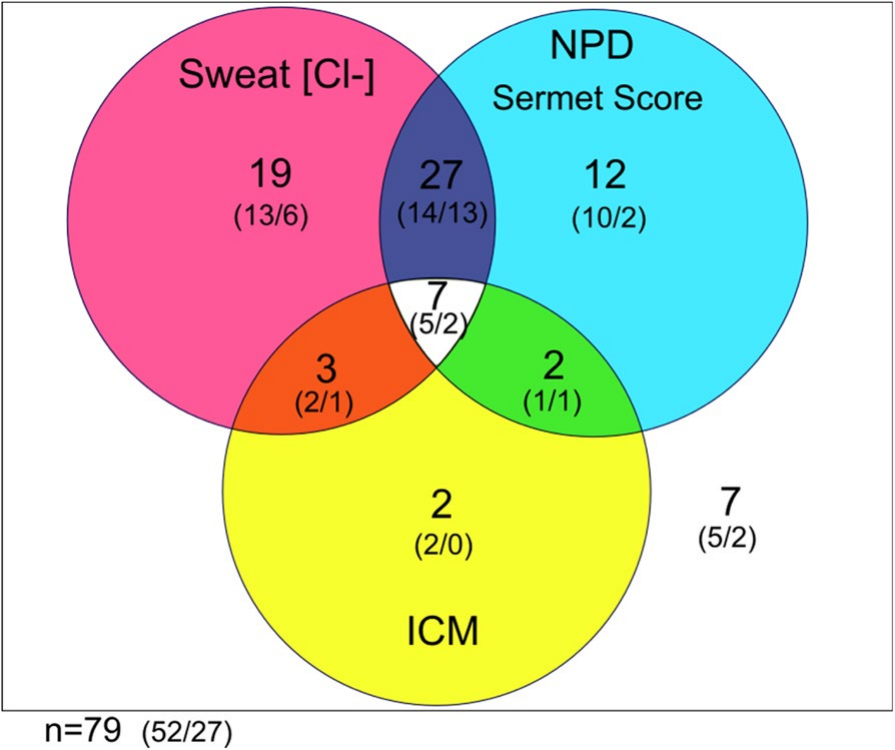
Venn diagram of the response of CFTR biomarkers of pwCF with one or two F508del alleles to 8–16 weeks of treatment with ELX/TEZ/IVA compared to baseline. The Venn diagram indicates the number of participants who normalized the response to the respective biomarker. A study participant showed a normalized response in the respective biomarker if SCC was below 60 mmol/L in QPIT [63], NPD had revealed a Sermet score of more than 0.27 [87], and the chloride secretory responses in the ICM had reached 70% of the mean normal values for at least two of three criteria, i.e. the response to activation with forskolin/IBMX, response to carbachol or the sum of the two responses [85, 91, 92]. Of the 79 study participants, 52 pwCF had been modulator naïve at baseline and 27 pwCF were administered combination therapy with tezacaftor and ivacaftor for at least six months. The numbers in brackets indicate modulator-naïve (left) and TEZ/IVA-positive pwCF at baseline (right) (Source: The authors’ Modulate-CF DZL consortium Berlin, Gießen, Hannover, Heidelberg [157])

**Fig. 2:**
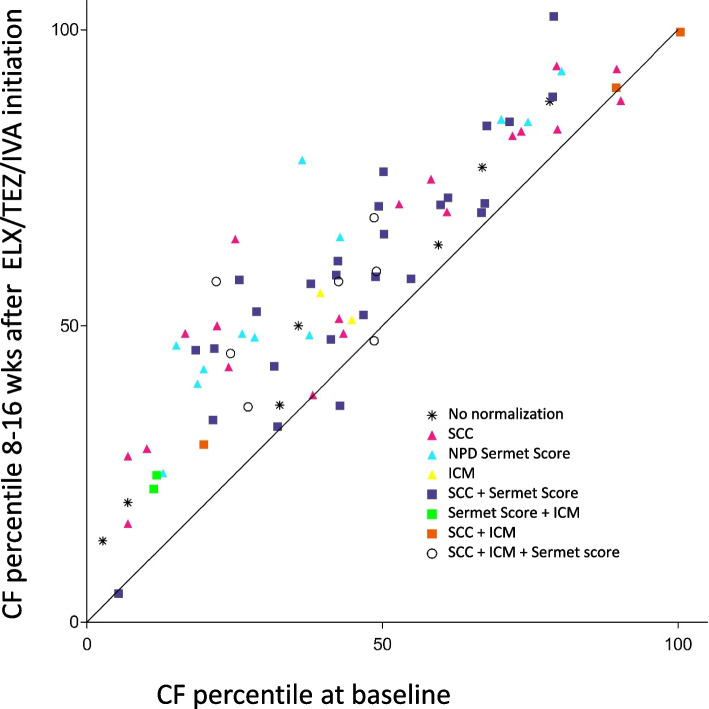
Personal clinical outcome and response to CFTR biomarkers of pwCF with one or two F508del alleles to 8–16 weeks of treatment with ELX/TEZ/IVA compared to baseline. Patients’ BMI and FEV1 values were mapped onto the age- and gender-corrected percentile distribution of the 2013 edition of the CF European registry [209]. Next, the FEV1 and BMI percentiles were combined into the CF disease percentile, which gives equal weight to lung function and anthropometry [211]. The figure shows for each of the 79 study participants the coordinate of CF disease percentiles at baseline and after 8–16 weeks exposure to ELX/TEZ/IVA. The diagonal separates individuals with improved or worsened percentile ranks upon initiation of ELX/TEZ/IVA. The individual’s normalization of CFTR biomarkers (defined as in Fig. 1) is indicated by symbol: asterisk, no normalization; red triangle, solely SCC [63]; blue triangle: solely NPD Sermet score [87]; yellow triangle, solely ICM [85, 91, 92]; purple square, SCC and NPD Sermet score; green square, NPD Sermet score and ICM; brown square; SCC and ICM; open circle, SCC + NPD Sermet score + ICM (Source: The authors’ Modulate-CF DZL consortium Berlin, Gießen, Hannover, Heidelberg [157])

The authors’ consortium ‘Modulate-CF’ in the German Center of Lung Research (DZL) examined the effects of ELX/TEZ/IVA on CFTR function in pwCF with one or two F508del alleles in three biomarkers, i.e. QPIT, NPD and ICM [157]. The median decrease of SCC of 48.5 mmol/L matched with that of the pooled data of the 21 studies (Table 1). 8–16 weeks after initiation of ELX/TEZ/IVA, CFTR function improved in nasal respiratory and intestinal epithelia to a median level close to half of normal [157]. However, the responses of the individual patients were highly variable. The Venn diagram in Fig. 1 shows the numbers of patients who developed a normal response in none, one, two or three CFTR biomarkers. Seven of the 79 study participants remained in the CF range for all three biomarkers and again seven patients achieved CFTR activity in the normal range for all three biomarkers. 33 and 32 study participants normalized in one or two biomarkers, respectively (Fig. 1). We were curious to know how these mixed responses in the basic defect of sweat gland, airway and intestine were associated with the clinical outcome in lung function and anthropometry, the key endpoints of the clinical trials. Based on registry data of European CF populations in the pre-modulator era as reference [209, 210], the FEV1 and BMI data of each patient were converted into age- and gender-corrected disease-specific percentiles [211]. The paired CF percentiles at baseline and after initiation of ELX/TEZ/IVA were mapped for each study participant on the two-dimensional percentile plot shown in Fig. 2. Normalization of CFTR biomarkers is indicated by colored symbols. More than 90% of study participants improved their CF percentile during the first 8 to 16 weeks of triple modulator therapy irrespectively of whether they were affected from mild or severe CF disease (Fig. 2). The normalization of SCC, NPD and/or ICM scattered over the whole range of disease percentiles (Fig. 2). In other words, we observed personal signatures of the normalization of the basic defect in sweat gland, airway and intestine that were not associated with the severity of CF disease at baseline and its improvement during the first weeks of treatment with ELX/TEZ/IVA. Restoration of CFTR function was diverse and not predictive for clinical outcome in the individual patient. For example, the patient with the largest absolute gain of ppFEV1 in our cohort exhibited the smallest change in sweat chloride. In summary, the individual responses of basic defect and clinical outcome measures were heterogeneous upon initiation of ELX/TEZ/IVA.

Despite being diverse, almost all responses to ELX/TEZ/IVA in the DZL cohort reflected improvements of CFTR function and general condition. Thus, we were taken by surprise to learn that this positive trend was not replicated by the β-adrenergic sweat secretion assay in a subgroup of the DZL cohort [57]. Triple therapy with ELX/TEZ/IVA normalized SCC, but gained only 4% of median wild-type β-adrenergic sweat rate [57]. β-adrenergic sweat stimulation in the coil is apparently more stringent in its requirement for CFTR activity than chloride reabsorption in the duct measured by the sweat test, because CFTR is only a minor component in the secretory coil and becomes the rate-limiting step of chloride secretion upon ß-adrenergic stimulation [52]. On the other hand, CFTR-mediated bicarbonate secretion in the kidney is more tolerant to compromised CFTR function than CFTR-mediated secretion and reabsorption of chloride in the sweat gland [116]. Bicarbonate excretion was close to normal for pwCF carrying F508del and a residual function mutation and treatment of F508del homozygotes with ELX/TEZ/IVA increased bicarbonate excretion to about 70% of healthy controls [113, 114].

Diversity of the response to ELX/TEZ/IVA in pwCF with one or two F508del alleles should reflect the impact of genetic and environmental modifiers on clinical outcome. F508del CFTR is a class II mutant defective in posttranslational processing and trafficking [19, 101, 102]. The members of the F508del interactome are thus the prime candidates for modifiers of corrector-mediated rescue of F508del CFTR. The amount of the thermolabile F508del CFTR protein in the apical epithelial membrane varies among pwCF and their organs and cells [101–105] and thus is was not a surprise to learn that the ELX/TEZ/IVA-mediated rescue of processing and targeting of F508del CFTR in the rectal epithelium differed substantively among the study participants of the DZL cohort [212]. Likewise, the rectal organoids of F508del homozygous donors showed personalized transcriptome profiles in presence and absence of ELX/TEZ/IVA [213]. Correspondingly, the profile of the top differentially expressed genes in the nasal epithelial transcriptome predicted with about 85% accuracy the individual patient’s improvement of FEV1 and BMI upon ELX/TEZ/IVA initiation [214]. The BIH consortium collected nasal swabs from children with CF and at least one F508del allele aged 6–11 years at baseline and three months after initiation of ELX/TEZ/IVA [73]. Compared to healthy children, CFTR-positive cells were decreased in epithelial basal, club and goblet cells and were restored by ELX/TEZ/IVA therapy to nearly healthy levels. Single cell cDNA sequencing revealed that ELX/TEZ/IVA partially restored the impaired innate immune response in epithelial cells and reduced the pro-inflammatory status of immune cells in the CF nasal mucosa [73]. This scRNA-seq study on airway epithelial and immune cells demonstrated for the first time that highly efficient triple CFTR modulator therapy might restore epithelial homeostasis and host defense in CF airways, particularly if treatment is started in children with preserved lung function [73].

### CFTR biomarker response to non-F508del *CFTR* genotypes

EMA had approved the therapy with ELX/TEZ/IVA for the 90% of pwCF in Europe aged 2 years or more who are harboring at least one F508del allele. Besides F508del, screening of 605 *CFTR* sequence variants in recombinant Fischer Rat Thyroid (FRT) cells identified further 496 variants (82%) that respond with more than 10% normal CFTR chloride transport activity to ELX/TEZ/IVA in vitro [215]. Thus, ELX/TEZ/IVA treatment should result in clinical benefit for almost all pwCF who do not carry two loss-of-function class I mutations. In the USA, the FDA has already approved 177 of these 605 variants for ELXTEZ/IVA therapy targeting 5.2% of the CF population in the US patient registry [216]. Prior to the expansion of the label in April 2025 by EMA (see below), off-label prescription of ELX/TEZ/IVA for patients with non-F508del *CFTR* genotypes in Europe has depended on case-by-case negotiations between CF physician and health insurance company. To identify the individuals who probably will gain clinical benefit from permanent therapy with this costly medication, it has become a common procedure to monitor CFTR activity with biomarkers during an initial pilot phase.

Table 2 provides an overview of published case reports of ‘theratyping’ of patient-derived organoids or epithelial cell cultures [76, 217–229]. Of 98 examined specimens from CF donors, 14 samples showed no extra CFTR activity upon exposure to ELX/TEZ/IVA and 61 samples showed a strong response to ELX/TEZ/IVA in the healthy range or in the range observed for CF donors with one or two F508alleles [217, 218]. The remaining 23 samples exhibited CFTR activity that was below the range for the F508del reference. Thus, 85% of samples from pwCF with non-F508del *CFTR* genotypes were responsive to triple modulator therapy.

**Table 2:** Theratyping: CFTR biomarker response of non-F508 del *CFTR* genotypes to ELX/TEZ/IVA

A. Rectal organoids
a. FIS assay [217–225]
• Response of genotype in the range of healthy or F508 del/F508 del or F508 del/class I mutation genotypes
G85E/A561E; G85E/I1234 V; G85E/W1282X; G85E/N1303K; G85E/N1303K; G85E/CFTRdele17a- 18; G85E/1677delTA; E92K/E92K; R117L;L997F/[R117L;L997F]; Q220X/A1006E; Q220X/A1006E; Q220X/R1066C; R334W/R764X; R334W/N1303K; [R334W;Q378X]/[R334W;Q378X]; R347P/CFTRdele17a- 18; G461E/N1303K; [L467F;F508del]/F508del; G542X/711 + 3 A > G; A559T/A559T; Q1012P/N1303K; R1066C/N1303K; R1066C/R1066H; R1066H/CFTRdele2,3; L1077P/N1303K; S1159F/S1159F; W1282X/N1303K; N1303K/N1303K; N1303K/N1303K; N1303K/2143delT; N1303K/2143delT; N1303K/3821delT; N1303K/3121 - 1 A > G; N1303K/3849 + 10kbC > T; 991del5/3849 + 10kbC > T; 3849 + 10kbC > T/3849 + 10kbC > T
• Response of genotype in the intermediate range
G85E/N1303K; R553X/3272–26 A > G; E585X/N1303K; N1303K/G542X; N1303K/W1282X; N1303K/W1282X; N1303K/N1303K; N1303K/N1303K; N1303K/N1303K; N1303K/N1303K; N1303K/N1303K; N1303K/N1303K; N1303K/4010delTATT; L1335P/L1335P; 1898 + 5G > T/3272 - 26 A > G; 2043delG/4382delA
• No response of genotype
M1V/N1303K; E60X/4015delATTT; R347P/L571S; G550X/N1303K; L927P/W1282X; W1282X/N1303K; N1303K/2184insA; 711 + 1G > T/2789 + 5G > A
b. Ussing chamber measurements of chloride secretion in organoid-derived rectal epithelial monolayers [219, 222, 225, 226]
• Response of genotype in the range of healthy or F508del/F508del or F508del/class I mutation genotypes
A559T/A559T; S737F/W1282X; S737F/CFTRdele22 - 24
• Response of genotype in the intermediate range R347P/R347P; T465N/Q39X
• No response of genotype L227R/L227R
c. Western immunoblot of CFTR protein [219, 222, 225, 226] Band C present: T465 N/Q39X; A559T/A559T; W57G/A234D; R347P/R347P
B. Nasal brushings
a. FIS assay [222, 227]
• Response of genotype in the range of healthy or F508del/F508del or F508del/class I mutation genotypes
W57G/A234D; L1077P/L1077P; L1077P/W1282X; L1077P/R1066 C
b. Ussing chamber measurements of chloride secretion in airway-liquid interface cultures [76, 219, 222, 223, 226, 227, 228, 229]
• Response of genotype in the range of healthy or F508del/F508del or F508del/class I mutation genotypes
L1077P/L1077P; L1077P/W1282X; L1077P/R1066C; N1303K/N1303K; W57G/A234D
• Response of genotype in the intermediate range
G85E/G85E; G1244E/G1244E*; G1244E/1717–1 G-A*; G1244E/G542X*; N1303K/N1303K
• No response of genotype
[L467F;F508del]/G542X; [L467F;F508del]/E585X; W1282X/W1282X; 2184 AA > G/3892delTT
*poor response to potentiator IVA, but strong response to potentiator apigenin
c. Western immunoblot of CFTR protein [226–228]
Band C absent: W1282X/W1282X; Band C present: W57G/A234D; L1077P/L1077P; L1077P/W1282X; G1244E/G1244E; G1244E/1717-1 G-A; G1244E/G542X

NPD and ICM have been rarely applied to test the effect of ELX/TEZ/IVA on CFTR activity. The authors examined a few patients with these biomarkers. Two individuals who are homozygous for G85E or N1303 K showed a response of 32% and 5% of normal in the NPD and of 29% and 15% in the ICM, respectively [76]. Two brothers who are compound heterozygous for class I donor and acceptor splice mutations that affect the same exon, presented an unusual response to ELX/TEZ/IVA [230]. Both patients showed no improvement in QPIT, NPD and ICM and clinical outcomes, but their CFTR-mediated β-adrenergic sweat rate normalized into the healthy range. To put this surprising finding into perspective, the reader may be reminded that pwCF with one or two F508del alleles will always improve in QPIT, NPD and ICM upon initiation of ELX/TEZ/IVA (see Fig. 1), but only about 10% of pwCF will gain a β-adrenergic sweat rate in the normal range after introduction of triple therapy [57].

The class II mutation N1303K present in 43 of 196 analyzed alleles (Table 2) received considerable attention of the ‘theratyping’ community. Located in the second nucleotide binding domain NBD2, N1303K CFTR is distinct from F508del CFTR in its channel properties and its pathway of posttranslational processing and trafficking [231–236]. Since N1303K CFTR was non-responsive to ELX/TEZ/IVA in recombinant FRT cells in vitro, N1303K was not included into the FDA-approved label. However, theratyping of patient-derived organoids and cell cultures with one or two N1303K alleles uncovered a broad spectrum of no (9%), intermediate (47%) or F508del-like responses (44%) to ELX/TEZ/IVA (Table 2). Based on the in vitro experimental evidence that N1303K CFTR showed activation with ELX/TEZ/IVA in their personal organoids, numerous patients commenced treatment with ELX/TEZ/IVA in the frame of clinical trials [223, 237, 238]. Study participants significantly improved in ppFEV1, CFQ-R and BMI, but showed no decrease in SCC and a variable personal response in NPD. Interestingly, rescue of N1303K and F508del CFTR was found to be enhanced under inflammatory conditions that are typical for CF airways [239, 240]. Sweat glands are not inflamed and thus the responses to CFTR modulators may be more limited than in inflamed CF airways or intestine. The supportive role of inflammation for the clinical efficacy of triple CFTR modulator therapy may also give a hint to understand why “changes in sweat Cl^−^ in individual pwCF are poorly predictive of clinical responses despite robust associations between sweat Cl^−^ responses and clinical efficacy on the population level” (Martina Gentzsch in ref. [239]).

The unanticipated clinical benefit of triple modulator therapy of the FRT-refractory N1303K mutation may not only reflect the broad spectrum of responses of individual patient’s CFTR mutant and its interactome to ELX/TEZ/IVA, but could also arise from off-target effects of the medication. Indeed, tezacaftor has been shown to alter the balance of the de-novo synthesis of sphingolipids by inhibition of sphingolipid delta- 4 desaturase that converts dihydroceramides into ceramides [241, 242]. Ceramide accumulates in the lungs of pwCF causing chronic inflammation, impairment of mucociliary clearance and susceptibility to bacterial infection [243–245]. Treatment with ELX/TEZ/IVA partially normalized the disturbed plasma sphingolipid profile in pwCF [246]. Tezacaftor does not only affect sphingolipid metabolism, but it also inhibits the SarcoEndoplasmic Reticulum Calcium ATPase (SERCA) [247]). Thereby tezacaftor and also elexacaftor normalize calcium homeostasis in F508 del-CFTR cells in a CFTR independent manner [248].

The critical anion secretion defect in CF is caused by diminished apical CFTR channel activity. As CFTR function is restored with the use of CFTR modulators, mechanisms that import chloride and bicarbonate across the basolateral membrane or generate bicarbonate within the cytosol may become rate limiting [240]. Rehman and colleagues now have shown that TNF-α + IL-17 treatment sensitizes primary CF airway epithelia to the beneficial effects of ELX/TEZ/IVA via p38 MAPK signaling [240]. The proinflammatory cytokines TNF-α + IL-17 increased the expression of CFTR and of Cl^–^ and HCO_3_^–^ importers and improved mucociliary clearance by lowering ASL viscosity.

These extra CFTR-independent benefits probably represent personal signatures. The variability of the transcriptome of F508del-homozygous airway epithelial cells was explained to 70% by differences between donors and only to 6% by the presence or absence of treatment with ELX/TEZ/IVA [249]. This strong Garrodian individuality of the CF airway transcriptome and its response to ELX/TEZ/IVA justify a posteriori the decision of the French Compassionate Program to prescribe ELX/TEZ/IVA for all pwCF with advanced lung disease irrespective of their *CFTR* genotype [250]. Meanwhile all pwCF without F508del variants who were living in France and aged 6 years or older have become eligible for a 4–6 week trial of ELX/TEZ/IVA [251]. Over half of the 516 treated pwCF responded to ELX/TEZ/IVA [251]. Among 360 participants with no FDA-approved variant and no previous CFTR modulator, 177 (49%) were responders; in responders, mean absolute change in SCC was − 20.5 mmol/L and ppFEV_1_ was 13.2 percentage points [251] consistent with the improvement of ppFEV1 seen in pwCF with one or two F508del alleles (see Table 1). CFTR potentiators and CFTR correctors have initially been developed for mutation-specific therapies in CF (reviewed in [247]), but the outcome of the expanded French Compassionate Program [251] tells us that efficient triple therapy with ELX/TEZ/IVA is potentially beneficial for almost all pwCF but the unfortunate ones who are carrying two non-responsive loss-of-function class I mutations. In other words, the Garrodian approach of individual theratyping paved the way to an Oslerian label for almost all *CFTR* genotypes.

Consistent with this conclusion, the efforts of the academia to show efficacy of ELX/TEZ/IVA for many non-F508del *CFTR* mutations have convinced the regulators to expand the label. On 04 April 2025, the European Medicines Agency (EMA) adopted extensions to the existing indications for ELX/TEZ/IVA to extend its use to all pwCF aged two years or more who carry at least one non-class I *CFTR* mutation. In other words, only the small group of pwCF who carry two class I mutations and hence do not produce CFTR protein will remain excluded from triple CFTR modulator therapy.

### Future directions

ELX/TEZ/IVA has improved the quality of life of pwCF under real-world conditions. However, the 5 years since approval are too short to conclude whether triple modulator therapy may halt the progression of CF lung disease in the long run. Airway dysbiosis [186, 192, 193, 196], inflammation [252–256] and unpleasant gastrointestinal symptoms [199, 257] persist – caveats we need to bear in mind. Moreover, late effects may emerge during life-long therapy. On the other hand, the younger the patient, the more SCC decreased [150, 179, 206] and the more epithelial homeostasis and host defense were restored [73] upon initiation of ELX/TEZ/IVA suggesting that we can expect maximal benefit of triple modulator therapy if we start treatment early in life in children with preserved lung function. This upcoming generation of pwCF will hopefully experience a rather normal life in childhood and adolescence that is not burdened anymore by extensive therapeutic measures and repeated hospitalization.

Conversely, today’s generation of CF adults who were modulator-naïve for decades, will remain compromised in their health by multiple primary and age-related secondary co-morbidities [7]. Likewise, by early 2025 CFTR modulators are still not a game changer for a large part of the CF patient population. First, access to the costly CFTR modulators is still not in place for eligible pwCF in many countries [258, 259]. Second, we need to establish and optimize dosage regimens for the unlucky few who are affected from relative or absolute contraindications such as severe hepatobiliary disease [260, 261] or treatment of infections with mycobacteria [262]. Third, a subset of patients – termed modulator-refractory CF – continues to experience two or or more pulmonary exacerbations per year requiring hospitalization or intravenous antibiotics, regardless of other modulator benefits [263]. Lastly, an at-risk subgroup of pwCF receiving ELX/TEZ/IVA has been identified [264, 265] who reported negative side effects in their mental well-being, which led to intermittent or definitive discontinuation of drug taking. These adverse neuropsychiatric effects are the most prevalent adverse drug reaction of CFTR modulator therapy [266]. The etiology is unknown. Participants of a workshop organized by the Cystic Fibrosis Foundation recommended that future studies should focus on understanding the role of CFTR in the nervous system, defining ELX/TEZ/IVA impacts in preclinical models [265].

ELX/TEZ/IVA pass the placental barrier and the lactating breast [267]. As demonstrated in the CF ferret, exposure to ELX/TEZ/IVA in utero may prevent fetal and postnatal pathologies associated with CF [268]. Consistent with these findings in the animal model, prenatal ELX/TEZ/IVA through a CF carrier mother and a mother with CF prevented meconium ileus [269] and retained a normal vas deferens in fetuses with CF [270], respectively. To develop ethical principles and consensus guidelines for care of reproductive-aged people on modulator therapy that is not based on accidental case reports, the CF community is urged to collect data in the next years [271] that characterize maternal, fetal and long-term offspring outcomes following CFTR modulator therapy use during pregnancy and breastfeeding [272].

Current CFTR biomarker studies focus on ELX/TEZ/IVA, but this scenario will change in near future. A novel triple therapy (VNZ triple) is available for pwCF in the US since January 2025. The corrector elexacaftor is exchanged by vanzacaftor and the potentiator ivacaftor by its deuterated analogue deutivacaftor [129]. Due to more favorable pharmacokinetics, VNZ triple only needs to be administered once daily. Three phase 3 clinical trials with more than 1,000 study participants tested VNZ triple vs. its comparator ELX/TEZ/IVA for 24 weeks. The absolute change of ppFEV1 from baseline through week 24 was not different between ELX/TEZ/IVA and VNZ triple [273, 274]. VNZ triple was superior to ELX/TEZ/IVA in proportion of participants achieving SCC < 60 mmol/L (86% vs. 77% in pwCF aged 12 years or more and 95% vs. 84% in pwCF aged 6–11 years) and in proportion of participants achieving SCC < 30 mmol/L (31% vs.23% in pwCF aged 12 years or more and 53% vs. 39% in pwCF aged 6–11 years) [273, 274]. Referring to the experience of the differential efficacy of ELX/TEZ/IVA on the N1303K mutant in sweat gland and airways [223, 237–240], the significance of the gain of CFTR activity in the sweat gland on clinical outcomes remains an open question that could be addressed in post-approval studies with more CFTR biomarkers and more sensitive clinical endpoints that had applied by the DZL consortium in the past [157, 171, 179].

So far, all approved CFTR modulators were developed and marketed by Vertex Pharmaceuticals. Other groups from academia and industry have yet not succeeded to bring any of their drugs to approval for treatment of pwCF. However, by 2019 Sionna Therapeutics was formed that has the mission to fully normalize CFTR function. One CFTR potentiator and five CFTR correctors that target locations in MSD2, MSD1, NBD1 or ICL4 are currently being studied in phase 1 or phase 2 clinical trials [275, 276]. By the time of writing, publicly available peer-reviewed reports of preclinical and clinical research with these compounds were still missing.

A further long running option for CF drugs that has not yet being materialized in clinical practice, are compounds that modify sodium or alternative chloride channels [277]. The activation of the Ca^2+^ activated channels TMEM16A [278] and SLC26A9 [279, 280] has been the major focus of research, but divergent modes of action and inapt localization in cells and organs sustain the long-lasting debate whether this CFTR-agnostic approach is beneficial for the treatment of CF [281–283].

The *CFTR* gene, its cDNA and mRNA transcripts are further targets for personalized therapies of CF [284]. Antisense oligonucleotide (ASO)-based exon skipping for splicing modulation has been developed for the 3849 + 10 kb C-to-T [285] splicing and the W1282X [286–288] nonsense mutations. The ASO drug SPL84 prevents the inclusion of a cryptic exon from the 3849 + 10 kb C-to-T allele and thus leads to an increase of correctly spliced CFTR mRNA and higher levels of functional protein [285]. Inhalation of SPL84 led to a broad distribution in cells and nuclei of mouse and monkey lungs [289] and was safe in healthy volunteers in a phase I study [290].

Besides ASOs, the knockdown of a key player in translation termination, i.e. the eukaryotic release factor 3a [291], and the engineering of suppressor tRNAs are promising strategies to tackle premature stop codons [292, 293]. Albers and colleagues designed a strategy that is based on altering native tRNAs into efficient suppressor tRNAs by individually fine-tuning their sequence to the physico-chemical properties of the amino acid that they carry [293]. The engineered suppressor tRNAs re-established expression and function of *CFTR* stop mutations in cell systems and CF patient-derived nasal epithelia and restored airway volume homeostasis [293]. Thereby the translation velocity of the sequence upstream of the premature stop codon critically modulated readthrough efficacy [294]. This treatment-response heterogeneity calls for personalized tRNA-based gene therapies in the future [292, 294]. Besides academia, Southern Research, a nonprofit contract research organization, is working on potential therapies for nonsense mutations [276].

The ‘universal strategy’ of gene therapy aims to offer treatment for all pwCF. Wild type *CFTR* gene, CFTR cDNA, CFTR mRNA or a correction system are delivered to the target of interest. Unless stem cells are manipulated, gene therapy allows no systemic therapy as with modulators, but will target one preferred organ such as the CF lung. Prime editing is the most recent addition to the portfolio of programmable gene-editing tools [295]. In contrast to CRISPR/cas genome correction by gene replacement [296], knock-in [297] or homology-directed repair [298], prime editors do not require the creation of double-strand DNA breaks, minimizing any unwanted on-target and off-target editing [295]. Its potential for curative CF gene editing therapy has recently been demonstrated in cell lines, organoids and primary cells for the L227R, N1303K and F508del mutations [299, 300]. The optimization of prime editing for the mutation of interest is not trivial. The reader is recommended to learn from the pioneers’ report about the multiple steps that were necessary to finally gain functional correction of F508del *CFTR* in airway epithelium [300].

Pulmonary delivery of nucleic acids to the CF lung epithelia has substantially advanced during the last 30 years and can now be executed by inhalation with lipid nanoparticles [298, 301, 302] or recombinant lentivirus [303, 304] or adeno-associated virus (AAV) [305]. Preclinical research currently focuses on full-length *CFTR* gene delivery, gene editing, phages as vectors and novel formulations [276]. The on-going phase 1 and phase 2 trials investigate aerosolized delivery of CFTR mRNA in lipid nanoparticles or of CFTR cDNA with AAV or lentivirus vectors [276, 303, 304]. To remain up-to-date of this fast-moving field, the reader may regularly consult the CFF website [276].

The UK Respiratory Gene Therapy Consortium whose scholars probably gained worldwide the most profound expertise for CF gene addition therapy within the last 20 years [306], will execute the first-in-human trial with a recombinant lentiviral vector in pwCF [303, 304, 307]. The third-generation lentiviral vector rSIV.F/HN carries a codon-optimized CpG-depleted CFTR cDNA, is pseudotyped for receptors on the apical surface of airway epithelia and has been engineered to minimize the risk of insertional oncogenesis, the major safety concern of lentiviral vectors [303, 304]. Lentiviral vectors integrate into the genome of transduced cells so that the cDNA insert can be expressed throughout the lifetime of the cell. The lentiviral vectors can incorporate comparably large inserts such as the full-length CFTR cDNA and evoke only minor inflammatory host responses. Based on the UK consortium’s meticulously performed preclinical work on vector design to optimize safety and efficacy, we can expect the maximal possible benefit of gene therapy with a viral vector for pwCF that is feasible with current knowledge and technology.

## Conclusions

All nucleic acid – based approaches of the last 35 years to cure CF succeeded to demonstrate the proof of principle in cells and animal models, but failed to correct CF in humans. Despite the tremendous improvements of tools to edit any human gene in the genome with high precision and minimal off-target effects, the formulation, delivery, and transduction of vector and its long-term correctly regulated gene expression remain challenging problems. The CF community will be anxious to learn whether Lenticlair™ of the UK Respiratory Gene Therapy Consortium [303, 304] will make a difference and will bring for the first time mutation-agnostic gene addition therapy to the CF patient. The optimal outcome would be the restoration of CFTR function in the lung mimicking the in vivo distribution of tasks between basal cells, secretory cells and ionocytes to secrete and absorb chloride and bicarbonate [67–72].

However, even the best possible outcome will correct function in just one major organ of the systemic disorder CF. Thus, the success of triple CFTR modulator therapy with small molecules cannot be valued highly enough as it has brought to almost all pwCF the partial restoration of CFTR function in virtually all affected organs. When researchers in the mid-1990 s started to set up high-throughput assays to mitigate the basic defect in CF, they pursued the Garrodian approach to develop therapies for a specific class of mutations or just the major mutation F508del. Theratyping of non-F508del mutations and the uniform treatment of all *CFTR* genotypes by the expanded French Compassionate Program [76, 217–229, 250, 251] have now taught us that ELX/TEZ/IVA will improve the function of virtually all CFTR mutants if CFTR protein is synthesized in sufficient amounts. The researchers at Vertex have screened more than a million compounds in their high-throughput assays, 12 molecules of which were later examined in clinical trials with pwCF [8]. This highly selected set of molecules apparently recognizes the critical Achilles heels of proper processing, trafficking and function of the CFTR protein. Recombinant ELX/TEZ/IVA-bound F508del CFTR showed wild-type conformations in cryo-EM [146]. The binding sites for elexacaftor, tezacaftor and ivacaftor are not in close proximity to the position of phenylalanine 508 in NBD1. Thus, ELX/TEZ/IVA induces not only in F508del CFTR, but also in many more missense mutants close-to-normal conformations. Guided by the Garrodian approach of mutation-specific therapy, Oslerian-type correctors and potentiators emerged from the evolutionary race of small molecules in the high-throughput assays that fit (almost) all mutants. EMA’s recent approval of ELX/TEZ/IVA for all pwCF who produce mutant CFTR protein rewards the efforts of the CF community of the last five years to demonstrate the efficacy of triple therapy for non-F508del mutations.

On the other hand, we observe substantial heterogeneity of the responses of the individual patient in CFTR biomarkers and clinical outcomes (cf. Figures 1, 2). The biomarkers demonstrate normalization of CFTR activity on the population level, but fail to predict the association between CFTR activity and clinical outcome. The next years will tell us whether early treatment of children with minimal pathology of the affected organs will generate more coherent presentations of basic defect and of clinical phenotype. During pre-modulator times, CF disease showed personalized signatures that were more shaped by genetic modifiers, infection, inflammation, socioeconomic status and therapeutic intervention than by the disease-causing *CFTR* mutations [65, 66]. The authors are curious which types of personal signatures of CF disease will emerge during permanent treatment with efficient CFTR modulators. The Garrodian-type CFTR biomarkers of the next generation should address the long-term course of airway and gut microbiome [196, 211, 308], host defense, epithelial homeostasis [73] and multiorgan metabolism [117].

## Data Availability

No datasets were generated or analysed during the current study.

## Abbreviations

AAV: Adeno-associated virus
ABPA: Allergic bronchopulmonary aspergillosis
ALI: Airway-liquid interface
ASL: Airway surface liquid
ASO: Antisense oligonucleotide
BIH: Berlin Institute of Health
CAVD: Congenital absence of the vas deferens
CF: Cystic fibrosis
CFF: Cystic Fibrosis Foundation
CFTR: Cystic fibrosis transmembrane conductance regulator
CFTR-RD: CFTR-related disorder
DIDS: 4,4'-Diisothiocyanostilbene-2, 2'-disulfonic acid
DVA: Deutivacaftor
DZL: German Center of Lung Research
ELX: Elexacaftor
EM: Electron microscopy
EMA: European Medicines Agency
ER: Endoplasmic reticulum
FDA: US Food and Drug Administration
FIS: Forskolin-induced swelling
FRT: Fischer Rat Thyroid
HGVS: Human genome variant sequence
IBMX: Isobutylmethylxanthin
ICM: Intestinal current measurements
ICL4: Intracellular loop 4
IL: Interleukin
IQR: Intraquartile range
IVA: Ivacaftor
LUM: Lumacaftor
MAPK: Mitogen-activated protein kinase
MSD: Membrane-spanning domain
NBD: Nucleotide-binding domain
NPD: Nasal transepithelial potential difference
PI: Exocrine pancreatic insufficiency
PS: Exocrine pancreatic sufficiency
PTC: Premature termination codon
pwCF: People with cystic fibrosis
QPIT: Quantitative pilocarpine iontophoresis sweat test
RD: Regulatory domain
ROMA: Rectal organoid morphology analysis
SCC: Sweat chloride concentration
scRNA-seq: Single cell RNA sequencing
SERCA: SarcoEndoplasmic Reticulum Calcium ATPase
TEZ: Tezacaftor
TM: Transmembrane segment
TNF-α: Tumor necrosis factor
VAN: Vanzacaftor
VUS: Variant of unclear clinical significance

## References

[1] Childs B (1999) Genetic Medicine: A Logic of Disease. Johns Hopkins University Press, Baltimore

[2] Mall MA, Burgel PR, Castellani C, Davies JC, Salathe M, Taylor-Cousar JL (2024) Cystic fibrosis. Nat Rev Dis Primers 10:53. 10.1038/s41572-024-00538-6 39117676 10.1038/s41572-024-00538-6

[3] Grasemann H, Ratjen F (2023) Cystic Fibrosis. N Engl J Med 389:1693–1707. 10.1056/NEJMra2216474 37913507 10.1056/NEJMra2216474

[4] Guo J, King I, Hill A (2024) International disparities in diagnosis and treatment access for cystic fibrosis. Pediatr Pulmonol 59:1622–1630. 10.1002/ppul.26954 38558542 10.1002/ppul.26954

[5] Bobadilla JL, Macek M Jr, Fine JP, Farrell PM (2002) Cystic fibrosis: a worldwide analysis of CFTR mutations–correlation with incidence data and application to screening. Hum Mutat 19:575–606. 10.1002/humu.10041 12007216 10.1002/humu.10041

[6] Gustafsson JK, Ermund A, Ambort D, Johansson ME, Nilsson HE, Thorell K, Hebert H, Sjövall H, Hansson GC (2012) Bicarbonate and functional CFTR channel are required for proper mucin secretion and link cystic fibrosis with its mucus phenotype. J Exp Med 209:1263–1272. 10.1084/jem.20120562 22711878 10.1084/jem.20120562 PMC3405509

[7] Felipe Montiel A, Fernández AÁ, Amigo MC, Traversi L, Clofent Alarcón D, Reyes KL, Polverino E (2024) The ageing of people living with cystic fibrosis: what to expect now? Eur Respir Rev 33:240071. 10.1183/16000617.0071-2024 39477350 10.1183/16000617.0071-2024 PMC11522972

[8] Tümmler B (2024) Geschichtlicher Abriss der Mukoviszidose. Monatsschr Kinderheilkd 172:487–493. 10.1007/s00112-024-01960-6

[9] Dittrich AM (2024) Mukoviszidose – eine Erfolgsgeschichte der Kinderheilkunde. Monatsschr Kinderheilkd 172:494–503 (2024). 10.1007/s00112-023-01796-6.

[10] Nährlich L, Burkhart M, Wosniok J (2024) Deutsches Mukoviszidose Register. Berichtsband 2023. www.muko.info/fileadmin/user_upload/was_wir_tun/register/berichtsbaende/berichtsband_2023.pdf

[11] Tümmler B (2023) Post-approval studies with the CFTR modulators Elexacaftor-Tezacaftor-Ivacaftor. Front Pharmacol 14:1158207. 10.3389/fphar.2023.1158207 37025483 10.3389/fphar.2023.1158207 PMC10072268

[12] Anderson MP, Gregory RJ, Thompson S, Souza DW, Paul S, Mulligan RC, Smith AE, Welsh MJ (1991) Demonstration that CFTR is a chloride channel by alteration of its anion selectivity. Science 253:202–205. 10.1126/science.1712984 1712984 10.1126/science.1712984

[13] Linsdell P, Tabcharani JA, Hanrahan JW (1997) Multi-Ion mechanism for ion permeation and block in the cystic fibrosis transmembrane conductance regulator chloride channel. J Gen Physiol 110:365–377. 10.1085/jgp.110.4.365 9379169 10.1085/jgp.110.4.365 PMC2229374

[14] Tang L, Fatehi M, Linsdell P (2009) Mechanism of direct bicarbonate transport by the CFTR anion channel. J Cyst Fibros 8:115–121. 10.1016/j.jcf.2008.10.004 19019741 10.1016/j.jcf.2008.10.004

[15] Van Goor F, Hadida S, Grootenhuis PD, Burton B, Cao D, Neuberger T, Turnbull A, Singh A, Joubran J, Hazlewood A, Zhou J, McCartney J, Arumugam V, Decker C, Yang J, Young C, Olson ER, Wine JJ, Frizzell RA, Ashlock M, Negulescu P (2009) Rescue of CF airway epithelial cell function in vitro by a CFTR potentiator, VX-770. Proc Natl Acad Sci U S A 106:18825–18830. 10.1073/pnas.0904709106 19846789 10.1073/pnas.0904709106 PMC2773991

[16] Brindani N, Gianotti A, Giovani S, Giacomina F, Di Fruscia P, Sorana F, Bertozzi SM, Ottonello G, Goldoni L, Penna I, Russo D, Summa M, Bertorelli R, Ferrera L, Pesce E, Sondo E, Galietta LJV, Bandiera T, Pedemonte N, Bertozzi F (2020) Identification, structure-activity relationship, and biological characterization of 2,3,4,5-tetrahydro-1*H*-pyrido[4,3-*b*] indoles as a novel class of CFTR potentiators. J Med Chem 63:11169–11194. 10.1021/acs.jmedchem.0c01050 32946228 10.1021/acs.jmedchem.0c01050 PMC8011931

[17] Phuan PW, Veit G, Tan JA, Finkbeiner WE, Lukacs GL, Verkman AS (2015) Potentiators of defective ΔF508-CFTR gating that do not interfere with corrector action. Mol Pharmacol 88:791–799. 10.1124/mol.115.099689 26245207 10.1124/mol.115.099689 PMC4576684

[18] Van Goor F, Hadida S, Grootenhuis PD, Burton B, Stack JH, Straley KS, Decker CJ, Miller M, McCartney J, Olson ER, Wine JJ, Frizzell RA, Ashlock M, Negulescu PA (2011) Correction of the F508del-CFTR protein processing defect in vitro by the investigational drug VX-809. Proc Natl Acad Sci U S A 108:18843–18848. 10.1073/pnas.1105787108 21976485 10.1073/pnas.1105787108 PMC3219147

[19] Okiyoneda T, Veit G, Dekkers JF, Bagdany M, Soya N, Xu H, Roldan A, Verkman AS, Kurth M, Simon A, Hegedus T, Beekman JM, Lukacs GL (2013) Mechanism-based corrector combination restores ΔF508-CFTR folding and function. Nat Chem Biol 9:444–454. 10.1038/nchembio.1253 23666117 10.1038/nchembio.1253 PMC3840170

[20] Keating D, Marigowda G, Burr L, Daines C, Mall MA, McKone EF, Ramsey BW, Rowe SM, Sass LA, Tullis E, McKee CM, Moskowitz SM, Robertson S, Savage J, Simard C, Van Goor F, Waltz D, Xuan F, Young T, Taylor-Cousar JL; VX16-445-001 Study Group (2018) VX-445-Tezacaftor-Ivacaftor in patients with Cystic Fibrosis and one or two Phe508del alleles. N Engl J Med 379:1612-1620. 10.1056/NEJMoa1807120 10.1056/NEJMoa1807120 PMC6289290 30334692

[21] Taylor-Cousar JL, Munck A, McKone EF, van der Ent CK, Moeller A, Simard C, Wang LT, Ingenito EP, McKee C, Lu Y, Lekstrom-Himes J, Elborn JS (2017) Tezacaftor-Ivacaftor in patients with Cystic Fibrosis homozygous for Phe508del. N Engl J Med 377:2013–2023. 10.1056/NEJMoa1709846 29099344 10.1056/NEJMoa1709846

[22] Rowe SM, Daines C, Ringshausen FC, Kerem E, Wilson J, Tullis E, Nair N, Simard C, Han L, Ingenito EP, McKee C, Lekstrom-Himes J, Davies JC (2017) Tezacaftor-Ivacaftor in residual-function heterozygotes with Cystic Fibrosis. N Engl J Med 377:2024–2035. 10.1056/NEJMoa1709847 29099333 10.1056/NEJMoa1709847 PMC6472479

[23] Heijerman HGM, McKone EF, Downey DG, Van Braeckel E, Rowe SM, Tullis E, Mall MA, Welter JJ, Ramsey BW, McKee CM, Marigowda G, Moskowitz SM, Waltz D, Sosnay PR, Simard C, Ahluwalia N, Xuan F, Zhang Y, Taylor-Cousar JL, McCoy KS; VX17-445-103 Trial Group (2019) Efficacy and safety of the elexacaftor plus Tezacaftor plus ivacaftor combination regimen in people with cystic fibrosis homozygous for the F508del mutation: a double-blind, randomised, phase 3 trial. Lancet 394:1940-1948. 10.1016/S0140-6736(19)32597-8 10.1016/S0140-6736(19)32597-8 PMC7571408 31679946

[24] Middleton PG, Mall MA, Dřevínek P, Lands LC, McKone EF, Polineni D, Ramsey BW, Taylor-Cousar JL, Tullis E, Vermeulen F, Marigowda G, McKee CM, Moskowitz SM, Nair N, Savage J, Simard C, Tian S, Waltz D, Xuan F, Rowe SM, Jain R; VX17-445-102 Study Group (2019) Elexacaftor-Tezacaftor-Ivacaftor for Cystic Fibrosis with a single Phe508del allele. N Engl J Med 381:1809-1819. 10.1056/NEJMoa1908639 10.1056/NEJMoa1908639 PMC7282384 31697873

[25] Sutharsan S, McKone EF, Downey DG, Duckers J, MacGregor G, Tullis E, Van Braeckel E, Wainwright CE, Watson D, Ahluwalia N, Bruinsma BG, Harris C, Lam AP, Lou Y, Moskowitz SM, Tian S, Yuan J, Waltz D, Mall MA; VX18-445-109 study group (2022) Efficacy and safety of elexacaftor plus tezacaftor plus ivacaftor versus tezacaftor plus ivacaftor in people with cystic fibrosis homozygous for F508del-CFTR: a 24-week, multicentre, randomised, double-blind, active-controlled, phase 3b trial. Lancet Respir Med 10:267-277. 10.1016/S2213-2600(21)00454-9 10.1016/S2213-2600(21)00454-9 34942085

[26] Sutharsan S, Dillenhoefer S, Welsner M, Stehling F, Brinkmann F, Burkhart M, Ellemunter H, Dittrich AM, Smaczny C, Eickmeier O, Kappler M, Schwarz C, Sieber S, Naehrig S, Naehrlich L; German CF Registry of the Mukoviszidose e.V. and participating CF sites (2023) Impact of elexacaftor/tezacaftor/ivacaftor on lung function, nutritional status, pulmonary exacerbation frequency and sweat chloride in people with cystic fibrosis: real-world evidence from the German CF Registry. Lancet Reg Health Eur 32:100690. 10.1016/j.lanepe.2023.100690 10.1016/j.lanepe.2023.100690 PMC10405057 37554663

[27] Nichols DP, Paynter AC, Heltshe SL, Donaldson SH, Frederick CA, Freedman SD, Gelfond D, Hoffman LR, Kelly A, Narkewicz MR, Pittman JE, Ratjen F, Rosenfeld M, Sagel SD, Schwarzenberg SJ, Singh PK, Solomon GM, Stalvey MS, Clancy JP, Kirby S, Van Dalfsen JM, Kloster MH, Rowe SM; PROMISE Study group (2022) Clinical effectiveness of Elexacaftor/Tezacaftor/Ivacaftor in people with Cystic Fibrosis: A clinical trial. Am J Respir Crit Care Med 205:529-539. 10.1164/rccm.202108-1986OC 10.1164/rccm.202108-1986OC PMC8906485 34784492

[28] Lopez A, Daly C, Vega-Hernandez G, MacGregor G, Rubin JL (2023) Elexacaftor/tezacaftor/ivacaftor projected survival and long-term health outcomes in people with cystic fibrosis homozygous for F508del. J Cyst Fibros 22:607–614. 10.1016/j.jcf.2023.02.004 36849331 10.1016/j.jcf.2023.02.004

[29] CFTR2. Clinical and Functional Translation of CFTR (2025) US CF Foundation, Johns Hopkins University, The Hospital for Sick Children. https://cftr2.org/ Accessed 19 January, 2025.

[30] Claustres M, Thèze C, des Georges M, Baux D, Girodon E, Bienvenu T, Audrezet MP, Dugueperoux I, Férec C, Lalau G, Pagin A, Kitzis A, Thoreau V, Gaston V, Bieth E, Malinge MC, Reboul MP, Fergelot P, Lemonnier L, Mekki C, Fanen P, Bergougnoux A, Sasorith S, Raynal C, Bareil C (2017) CFTR-France, a national relational patient database for sharing genetic and phenotypic data associated with rare CFTR variants. Hum Mutat 38:1297-1315. 10.1002/humu.23276 10.1002/humu.23276 28603918

[31] CFTR France database (2017). https://cftr.chu-montpellier.fr/cgi-bin/home.cgi? Accessed 12 Dec 2024.

[32] Castellani C, De Boeck K, De Wachter E, Sermet-Gaudelus I, Simmonds NJ, Southern KW; ECFS Diagnostic Network Working Group (2022) ECFS standards of care on CFTR-related disorders: Updated diagnostic criteria. J Cyst Fibros 21:908-921. 10.1016/j.jcf.2022.09.011 10.1016/j.jcf.2022.09.011 36220763

[33] Sermet-Gaudelus I, Girodon E, Vermeulen F, Solomon GM, Melotti P, Graeber SY, Bronsveld I, Rowe SM, Wilschanski M, Tümmler B, Cutting GR, Gonska T (2022) ECFS standards of care on CFTR-related disorders: Diagnostic criteria of CFTR dysfunction. J Cyst Fibros 21:922–936. 10.1016/j.jcf.2022.09.005 36207272 10.1016/j.jcf.2022.09.005

[34] Simmonds NJ, Southern KW, De Wachter E, De Boeck K, Bodewes F, Mainz JG, Middleton PG, Schwarz C, Vloeberghs V, Wilschanski M, Bourrat E, Chalmers JD, Ooi CY, Debray D, Downey DG, Eschenhagen P, Girodon E, Hickman G, Koitschev A, Nazareth D, Nick JA, Peckham D, VanDevanter D, Raynal C, Scheers I, Waller MD, Sermet-Gaudelus I, Castellani C; ECFS Diagnostic Network Working Group (2024) ECFS standards of care on CFTR-related disorders: Identification and care of the disorders. J Cyst Fibros 23:590-602. 10.1016/j.jcf.2024.03.008 10.1016/j.jcf.2024.03.008 38508949

[35] De Wachter E, De Boeck K, Sermet-Gaudelus I, Simmonds NJ, Munck A, Naehrlich L, Barben J, Boyd C, Veen SJ, Carr SB, Fajac I, Farrell PM, Girodon E, Gonska T, Grody WW, Jain M, Jung A, Kerem E, Raraigh KS, van Koningsbruggen-Rietschel S, Waller MD, Southern KW, Castellani C; ECFS Diagnostic Network Working Group (2024) ECFS standards of care on CFTR-related disorders: Towards a comprehensive program for affected individuals. J Cyst Fibros 23:388-397. 10.1016/j.jcf.2024.01.012 10.1016/j.jcf.2024.01.012 38388234

[36] den Dunnen JT, Dalgleish R, Maglott DR, Hart RK, Greenblatt MS, McGowan-Jordan J, Roux AF, Smith T, Antonarakis SE, Taschner PE (2016) HGVS recommendations for the description of sequence variants: 2016 update. Hum Mutat 37:564–569. 10.1002/humu.22981 26931183 10.1002/humu.22981

[37] Zolin A, Adamoli A, Bakkeheim E, van Rens J (2024) ECFSPR Annual Report 2022. European Cystic Fibrosis Society. https://www.ecfs.eu/sites/default/files/Annual%20Report_2022_ECFSPR_20240603.pdf Accessed 19 Jan 2025.

[38] Riordan JR, Rommens JM, Kerem B, Alon N, Rozmahel R, Grzelczak Z, Zielenski J, Lok S, Plavsic N, Chou JL, Drumm ML, Iannuzzi MC, Collins FS, Tsui LC (1989) Identification of the cystic fibrosis gene: cloning and characterization of complementary DNA. Science 245:1066–1073. 10.1126/science.2475911 2475911 10.1126/science.2475911

[39] Hwang TC, Yeh JT, Zhang J, Yu YC, Yeh HI, Destefano S (2018) Structural mechanisms of CFTR function and dysfunction. J Gen Physiol 150:539–570. 10.1085/jgp.201711946 29581173 10.1085/jgp.201711946 PMC5881446

[40] Csanády L, Vergani P, Gadsby DC (2019) Structure, gating, and regulation of the CFTR anion channel. Physiol Rev 99:707–738. 10.1152/physrev.00007.2018 30516439 10.1152/physrev.00007.2018

[41] Zhang Z, Liu F, Chen J (2017) Conformational changes of CFTR upon phosphorylation and ATP binding. Cell 170:483-491.e8. 10.1016/j.cell.2017.06.041 28735752 10.1016/j.cell.2017.06.041

[42] Liu F, Zhang Z, Csanády L, Gadsby DC, Chen J (2017) Molecular structure of the human CFTR ion channel. Cell 169:85-95.e8. 10.1016/j.cell.2017.02.024. (PMID: 28340353) 28340353 10.1016/j.cell.2017.02.024

[43] Levring J, Terry DS, Kilic Z, Fitzgerald G, Blanchard SC, Chen J (2023) CFTR function, pathology and pharmacology at single-molecule resolution. Nature 616:606–614. 10.1038/s41586-023-05854-7 36949202 10.1038/s41586-023-05854-7 PMC10115640

[44] Linsdell P (2017) Architecture and functional properties of the CFTR channel pore. Cell Mol Life Sci 74:67–83. 10.1007/s00018-016-2389-5 27699452 10.1007/s00018-016-2389-5 PMC11107662

[45] Fay JF, Aleksandrov LA, Jensen TJ, Cui LL, Kousouros JN, He L, Aleksandrov AA, Gingerich DS, Riordan JR, Chen JZ (2018) Cryo-EM visualization of an active high open probability CFTR anion channel. Biochemistry 57:6234–6246. 10.1021/acs.biochem.8b00763 30281975 10.1021/acs.biochem.8b00763

[46] Levring J, Chen J. Structural identification of a selectivity filter in CFTR (2024) Proc Natl Acad Sci U S A 121:e2316673121. 10.1073/pnas.2316673121. 10.1073/pnas.2316673121 PMC10907310 38381791

[47] Welsh MJ, Smith AE (1993) Molecular mechanisms of CFTR chloride channel dysfunction in cystic fibrosis. Cell 73:1251–1254. 10.1016/0092-8674(93)90353-r 7686820 10.1016/0092-8674(93)90353-r

[48] Kristidis P, Bozon D, Corey M, Markiewicz D, Rommens J, Tsui LC, Durie P (1992) Genetic determination of exocrine pancreatic function in cystic fibrosis. Am J Hum Genet 50:1178–1184 1376016 PMC1682557

[49] Dekkers JF, Wiegerinck CL, de Jonge HR, Bronsveld I, Janssens HM, de Winter-de Groot KM, Brandsma AM, de Jong NW, Bijvelds MJ, Scholte BJ, Nieuwenhuis EE, van den Brink S, Clevers H, van der Ent CK, Middendorp S, Beekman JM (2013) A functional CFTR assay using primary cystic fibrosis intestinal organoids. Nat Med 19:939–945. 10.1038/nm.3201 23727931 10.1038/nm.3201

[50] Kim J, Farahmand M, Dunn C, Davies Z, Frisbee E, Milla C, Wine JJ (2016) Evaporimeter and bubble-imaging measures of sweat gland secretion rates. PLoS ONE 11:e0165254. 10.1371/journal.pone.0165254 27768743 10.1371/journal.pone.0165254 PMC5074501

[51] Wine JJ (2024) Calibrating sweat chloride levels to CFTR activity via ETI effects on CF subjects with one or two F508DEL mutations. J Cyst Fibros 23:1180–1184. 10.1016/j.jcf.2024.09.004 39406575 10.1016/j.jcf.2024.09.004

[52] Sato K, Sato F (1984) Defective beta adrenergic response of cystic fibrosis sweat glands in vivo and in vitro. J Clin Invest 73:1763–1771. 10.1172/JCI111385 6327771 10.1172/JCI111385 PMC437089

[53] Behm JK, Hagiwara G, Lewiston NJ, Quinton PM, Wine JJ (1987) Hyposecretion of beta-adrenergically induced sweating in cystic fibrosis heterozygotes. Pediatr Res 22:271–276. 10.1203/00006450-198709000-00007 2889182 10.1203/00006450-198709000-00007

[54] Quinton P, Molyneux L, Ip W, Dupuis A, Avolio J, Tullis E, Conrad D, Shamsuddin AK, Durie P, Gonska T (2012) β-adrenergic sweat secretion as a diagnostic test for cystic fibrosis. Am J Respir Crit Care Med 186:732–739. 10.1164/rccm.201205-0922OC 22859523 10.1164/rccm.201205-0922OC

[55] Salinas DB, Peng YH, Horwich B, Wee CP, Frisbee E, Maarek JM (2020) Image-based β-adrenergic sweat rate assay captures minimal cystic fibrosis transmembrane conductance regulator function. Pediatr Res 87:137–145. 10.1038/s41390-019-0503-8 31344706 10.1038/s41390-019-0503-8 PMC6962560

[56] Bergamini G, Tridello G, Calcaterra E, Ceri S, Tagliasacchi M, Bianchi F, Monti F, Masciadri A, Laudanna E, Peserico D, Sorio E, Esposito V, Leal T, Assael BM, Sorio C, Melotti P (2018) Ratiometric sweat secretion optical test in cystic fibrosis, carriers and healthy subjects. J Cyst Fibros 17:186–189. 10.1016/j.jcf.2017.12.003 29292091 10.1016/j.jcf.2017.12.003

[57] Pallenberg ST, Junge S, Ringshausen FC, Sauer-Heilborn A, Hansen G, Dittrich AM, Tümmler B, Nietert M (2022) CFTR modulation with elexacaftor-tezacaftor-ivacaftor in people with cystic fibrosis assessed by the β-adrenergic sweat rate assay. J Cyst Fibros 21:442–447. 10.1016/j.jcf.2021.10.005 34756683 10.1016/j.jcf.2021.10.005

[58] Gibson LE, Cooke RE (1959) A test for concentration of electrolytes in sweat in cystic fibrosis of the pancreas utilizing pilocarpine by iontophoresis. Pediatrics 23:545–549. 13633369

[59] Green A, Kirk J; Guidelines Development Group (2007) Guidelines for the performance of the sweat test for the diagnosis of cystic fibrosis. Ann Clin Biochem 44:25-34. 10.1258/000456307779596011 10.1258/000456307779596011 17270089

[60] Schulz IJ, Frömter E (1968) Mikropunktionsuntersuchungen an Schweißdrüsen von Mucoviscidose-Patienten und gesunden Versuchspersonen. In: Windorfer H, Stephan U (eds) Mucoviscidose. Georg Thieme Verlag, Stuttgart, Cystische Fibrose. II. Deutsches Symposion, pp 12–21

[61] Quinton PM (1983) Chloride impermeability in cystic fibrosis. Nature 301:421–422. 10.1038/301421a0 6823316 10.1038/301421a0

[62] Collaco JM, Blackman SM, Raraigh KS, Corvol H, Rommens JM, Pace RG, Boelle PY, McGready J, Sosnay PR, Strug LJ, Knowles MR, Cutting GR (2016) Sources of variation in sweat chloride measurements in Cystic Fibrosis. Am J Respir Crit Care Med 194:1375–1382. 10.1164/rccm.201603-0459OC 27258095 10.1164/rccm.201603-0459OC PMC5148144

[63] De Boeck K, Wilschanski M, Castellani C, Taylor C, Cuppens H, Dodge J, Sinaasappel M; Diagnostic Working Group (2006) Cystic fibrosis: terminology and diagnostic algorithms. Thorax 61:627-635. 10.1136/thx.2005.043539 10.1136/thx.2005.043539 PMC2104676 16384879

[64] McKone EF, Velentgas P, Swenson AJ, Goss CH (2015) Association of sweat chloride concentration at time of diagnosis and CFTR genotype with mortality and cystic fibrosis phenotype. J Cyst Fibros 14:580–586. 10.1016/j.jcf.2015.01.005 25660278 10.1016/j.jcf.2015.01.005

[65] Cutting GR (2015) Cystic fibrosis genetics: from molecular understanding to clinical application. Nat Rev Genet 16:45–56. 10.1038/nrg3849 25404111 10.1038/nrg3849 PMC4364438

[66] Stanke F, Becker T, Kumar V, Hedtfeld S, Becker C, Cuppens H, Tamm S, Yarden J, Laabs U, Siebert B, Fernandez L, Macek M Jr, Radojkovic D, Ballmann M, Greipel J, Cassiman JJ, Wienker TF, Tümmler B (2011) Genes that determine immunology and inflammation modify the basic defect of impaired ion conductance in cystic fibrosis epithelia. J Med Genet 48:24–31. 10.1136/jmg.2010.080937 20837493 10.1136/jmg.2010.080937 PMC3003880

[67] Stoltz DA, Meyerholz DK, Welsh MJ (2015) Origins of cystic fibrosis lung disease. N Engl J Med 372:351–362. 10.1056/NEJMra1300109 25607428 10.1056/NEJMra1300109 PMC4916857

[68] Okuda K, Dang H, Kobayashi Y, Carraro G, Nakano S, Chen G, Kato T, Asakura T, Gilmore RC, Morton LC, Lee RE, Mascenik T, Yin WN, Barbosa Cardenas SM, O’Neal YK, Minnick CE, Chua M, Quinney NL, Gentzsch M, Anderson CW, Ghio A, Matsui H, Nagase T, Ostrowski LE, Grubb BR, Olsen JC, Randell SH, Stripp BR, Tata PR, O’Neal WK, Boucher RC (2021) Secretory cells dominate airway CFTR expression and function in human airway superficial epithelia. Am J Respir Crit Care Med 203:1275–1289. 10.1164/rccm.202008-3198OC 33321047 10.1164/rccm.202008-3198OC PMC8456462

[69] Montoro DT, Haber AL, Biton M, Vinarsky V, Lin B, Birket SE, Yuan F, Chen S, Leung HM, Villoria J, Rogel N, Burgin G, Tsankov AM, Waghray A, Slyper M, Waldman J, Nguyen L, Dionne D, Rozenblatt-Rosen O, Tata PR, Mou H, Shivaraju M, Bihler H, Mense M, Tearney GJ, Rowe SM, Engelhardt JF, Regev A, Rajagopal J (2018) A revised airway epithelial hierarchy includes CFTR-expressing ionocytes. Nature 560:319–324. 10.1038/s41586-018-0393-7 30069044 10.1038/s41586-018-0393-7 PMC6295155

[70] Plasschaert LW, Žilionis R, Choo-Wing R, Savova V, Knehr J, Roma G, Klein AM, Jaffe AB (2018) A single-cell atlas of the airway epithelium reveals the CFTR-rich pulmonary ionocyte. Nature 560:377–381. 10.1038/s41586-018-0394-6 30069046 10.1038/s41586-018-0394-6 PMC6108322

[71] Lei L, Traore S, Romano Ibarra GS, Karp PH, Rehman T, Meyerholz DK, Zabner J, Stoltz DA, Sinn PL, Welsh MJ, McCray PB Jr, Thornell IM (2023) CFTR-rich ionocytes mediate chloride absorption across airway epithelia. J Clin Invest 133:e171268. 10.1172/JCI171268 37581935 10.1172/JCI171268 PMC10575720

[72] Luan X, Henao Romero N, Campanucci VA, Le Y, Mustofa J, Tam JS, Ianowski JP (2024) Pulmonary ionocytes regulate airway surface liquid pH in primary human bronchial epithelial cells. Am J Respir Crit Care Med 210:788–800. 10.1164/rccm.202309-1565OC 38573173 10.1164/rccm.202309-1565OC PMC11418883

[73] Loske J, Völler M, Lukassen S, Stahl M, Thürmann L, Seegebarth A, Röhmel J, Wisniewski S, Messingschlager M, Lorenz S, Klages S, Eils R, Lehmann I, Mall MA, Graeber SY, Trump S (2024) Pharmacological improvement of Cystic Fibrosis Transmembrane Conductance Regulator function rescues airway epithelial homeostasis and host defense in children with Cystic Fibrosis. Am J Respir Crit Care Med 209:1338–1350. 10.1164/rccm.202310-1836OC 38259174 10.1164/rccm.202310-1836OC PMC11146576

[74] Gentzsch M, Boyles SE, Cheluvaraju C, Chaudhry IG, Quinney NL, Cho C, Dang H, Liu X, Schlegel R, Randell SH (2017) Pharmacological rescue of conditionally reprogrammed Cystic Fibrosis bronchial epithelial cells. Am J Respir Cell Mol Biol 56:568–574. 10.1165/rcmb.2016-0276MA 27983869 10.1165/rcmb.2016-0276MA PMC5449492

[75] Balázs A, Millar-Büchner P, Mülleder M, Farztdinov V, Szyrwiel L, Addante A, Kuppe A, Rubil T, Drescher M, Seidel K, Stricker S, Eils R, Lehmann I, Sawitzki B, Röhmel J, Ralser M, Mall MA (2022) Age-related differences in structure and function of nasal epithelial cultures from healthy children and elderly people. Front Immunol 13:822437. 10.3389/fimmu.2022.822437 35296085 10.3389/fimmu.2022.822437 PMC8918506

[76] Graeber SY, Balázs A, Ziegahn N, Rubil T, Vitzthum C, Piehler L, Drescher M, Seidel K, Rohrbach A, Röhmel J, Thee S, Duerr J, Mall MA, Stahl M (2023) Personalized CFTR modulator therapy for *G85E* and *N1303K* homozygous patients with Cystic Fibrosis. Int J Mol Sci 24:12365. 10.3390/ijms241512365 37569738 10.3390/ijms241512365 PMC10418744

[77] Di A, Brown ME, Deriy LV, Li C, Szeto FL, Chen Y, Huang P, Tong J, Naren AP, Bindokas V, Palfrey HC, Nelson DJ (2006) CFTR regulates phagosome acidification in macrophages and alters bactericidal activity. Nat Cell Biol 8:933–944. 10.1038/ncb1456 16921366 10.1038/ncb1456

[78] Zhang S, Shrestha CL, Kopp BT (2018) Cystic fibrosis transmembrane conductance regulator (CFTR) modulators have differential effects on cystic fibrosis macrophage function. Sci Rep 8:17066. 10.1038/s41598-018-35151-7 30459435 10.1038/s41598-018-35151-7 PMC6244248

[79] Brinkert K, Hedtfeld S, Burhop A, Gastmeier R, Gad P, Wedekind D, Kloth C, Rothschuh J, Lachmann N, Hetzel M, Jirmo AC, Lopez-Rodriguez E, Brandenberger C, Hansen G, Schambach A, Ackermann M, Tümmler B, Munder A (2021) Rescue from *Pseudomonas aeruginosa* airway infection via stem cell transplantation. Mol Ther 29:1324–1334. 10.1016/j.ymthe.2020.12.003 33279724 10.1016/j.ymthe.2020.12.003 PMC7935663

[80] Weimann A, Dinan AM, Ruis C, Bernut A, Pont S, Brown K, Ryan J, Santos L, Ellison L, Ukor E, Pandurangan AP, Krokowski S, Blundell TL, Welch M, Blane B, Judge K, Bousfield R, Brown N, Bryant JM, Kukavica-Ibrulj I, Rampioni G, Leoni L, Harrison PT, Peacock SJ, Thomson NR, Gauthier J, Fothergill JL, Levesque RC, Parkhill J, Floto RA (2024) Evolution and host-specific adaptation of *Pseudomonas aeruginosa*. Science 385:eadi0908. 10.1126/science.adi0908. 10.1126/science.adi0908 PMC7618370 38963857

[81] Knowles M, Gatzy J, Boucher R (1981) Increased bioelectric potential difference across respiratory epithelia in cystic fibrosis. N Engl J Med 305:1489–1495. 10.1056/NEJM198112173052502 7300874 10.1056/NEJM198112173052502

[82] Standaert TA, Boitano L, Emerson J, Milgram LJ, Konstan MW, Hunter J, Berclaz PY, Brass L, Zeitlin PL, Hammond K, Davies Z, Foy C, Noone PG, Knowles MR (2004) Standardized procedure for measurement of nasal potential difference: an outcome measure in multicenter cystic fibrosis clinical trials. Pediatr Pulmonol 37:385–392. 10.1002/ppul.10448 15095320 10.1002/ppul.10448

[83] Schüler D, Sermet-Gaudelus I, Wilschanski M, Ballmann M, Dechaux M, Edelman A, Hug M, Leal T, Lebacq J, Lebecque P, Lenoir G, Stanke F, Wallemacq P, Tümmler B, Knowles MR (2004) Basic protocol for transepithelial nasal potential difference measurements. J Cyst Fibros 3(Suppl 2):151–155. 10.1016/j.jcf.2004.05.032 15463949 10.1016/j.jcf.2004.05.032

[84] European Cystic Fibrosis Society (ECFS) Diagnostic Working Group & Clinical Trials Network, Bronsveld I, Sermet-Gaudelus I (2013) Nasal potential difference (NPD) measurement for diagnosis and clinical trials in cystic fibrosis. NPD_EU01, version 1.7

[85] Minso R, Schulz A, Dopfer C, Alfeis N, Barneveld AV, Makartian-Gyulumyan L, Hansen G, Junge S, Müller C, Ringshausen FCC, Sauer-Heilborn A, Stanke F, Stolpe C, Tamm S, Welte T, Dittrich AM, Tümmler B (2020) Intestinal current measurement and nasal potential difference to make a diagnosis of cases with inconclusive *CFTR* genetics and sweat test. BMJ Open Respir Res 7:e000736. 10.1136/bmjresp-2020-000736 33020115 10.1136/bmjresp-2020-000736 PMC7537139

[86] Wilschanski M, Famini H, Strauss-Liviatan N, Rivlin J, Blau H, Bibi H, Bentur L, Yahav Y, Springer H, Kramer MR, Klar A, Ilani A, Kerem B, Kerem E (2001) Nasal potential difference measurements in patients with atypical cystic fibrosis. Eur Respir J 17:1208–1215. 10.1183/09031936.01.00092501 11491166 10.1183/09031936.01.00092501

[87] Sermet-Gaudelus I, Girodon E, Sands D, Stremmler N, Vavrova V, Deneuville E, Reix P, Bui S, Huet F, Lebourgeois M, Munck A, Iron A, Skalicka V, Bienvenu T, Roussel D, Lenoir G, Bellon G, Sarles J, Macek M, Roussey M, Fajac I, Edelman A (2010) Clinical phenotype and genotype of children with borderline sweat test and abnormal nasal epithelial chloride transport. Am J Respir Crit Care Med 182:929–936. 10.1164/rccm.201003-0382OC 20538955 10.1164/rccm.201003-0382OC

[88] Veeze HJ, Sinaasappel M, Bijman J, Bouquet J, de Jonge HR (1991) Ion transport abnormalities in rectal suction biopsies from children with cystic fibrosis. Gastroenterology 101:398–403. 10.1016/0016-5085(91)90017-f 2065916 10.1016/0016-5085(91)90017-f

[89] De Jonge HR, Ballmann M, Veeze H, Bronsveld I, Stanke F, Tümmler B, Sinaasappel M (2004) Ex vivo CF diagnosis by intestinal current measurements (ICM) in small aperture, circulating Ussing chambers. J Cyst Fibros 3(Suppl 2):159–163. 10.1016/j.jcf.2004.05.034 15463951 10.1016/j.jcf.2004.05.034

[90] Mall M, Hirtz S, Gonska T, Kunzelmann K (2004) Assessment of CFTR function in rectal biopsies for the diagnosis of cystic fibrosis. J Cyst Fibros 3(Suppl 2):165–169. 10.1016/j.jcf.2004.05.035 15463952 10.1016/j.jcf.2004.05.035

[91] Mall M, Bleich M, Schürlein M, Kühr J, Seydewitz HH, Brandis M, Greger R, Kunzelmann K (1998) Cholinergic ion secretion in human colon requires coactivation by cAMP. Am J Physiol 275:G1274–G1281. 10.1152/ajpgi.1998.275.6.G1274 9843763 10.1152/ajpgi.1998.275.6.G1274

[92] Bronsveld I, Mekus F, Bijman J, Ballmann M, Greipel J, Hundrieser J, Halley DJ, Laabs U, Busche R, De Jonge HR, Tümmler B, Veeze HJ (2000) Residual chloride secretion in intestinal tissue of deltaF508 homozygous twins and siblings with cystic fibrosis. The European CF Twin and Sibling Study Consortium. Gastroenterology 119:32–40. 10.1053/gast.2000.8524 10889152 10.1053/gast.2000.8524

[93] Yu K, Lujan R, Marmorstein A, Gabriel S, Hartzell HC (2010) Bestrophin-2 mediates bicarbonate transport by goblet cells in mouse colon. J Clin Invest 120:1722–1735. 10.1172/JCI41129 20407206 10.1172/JCI41129 PMC2860923

[94] Hoque KM, Woodward OM, van Rossum DB, Zachos NC, Chen L, Leung GP, Guggino WB, Guggino SE, Tse CM (2010) Epac1 mediates protein kinase A-independent mechanism of forskolin-activated intestinal chloride secretion. J Gen Physiol 135:43–58. 10.1085/jgp.200910339 20038525 10.1085/jgp.200910339 PMC2806414

[95] Duan T, Cil O, Thiagarajah JR, Verkman AS (2019) Intestinal epithelial potassium channels and CFTR chloride channels activated in ErbB tyrosine kinase inhibitor diarrhea. JCI Insight 4:e126444. 10.1172/jci.insight.126444 30668547 10.1172/jci.insight.126444 PMC6478423

[96] Linley J, Loganathan A, Kopanati S, Sandle GI, Hunter M (2014) Evidence that two distinct crypt cell types secrete chloride and potassium in human colon. Gut 63:472–479. 10.1136/gutjnl-2013-304695 23740188 10.1136/gutjnl-2013-304695

[97] Young PG, Levring J, Fiedorczuk K, Blanchard SC, Chen J (2024) Structural basis for CFTR inhibition by CFTR_inh_-172. Proc Natl Acad Sci U S A 121:e2316675121. 10.1073/pnas.2316675121 38422021 10.1073/pnas.2316675121 PMC10927578

[98] Hirtz S, Gonska T, Seydewitz HH, Thomas J, Greiner P, Kuehr J, Brandis M, Eichler I, Rocha H, Lopes AI, Barreto C, Ramalho A, Amaral MD, Kunzelmann K, Mall M (2004) CFTR Cl- channel function in native human colon correlates with the genotype and phenotype in cystic fibrosis. Gastroenterology 127:1085–1095. 10.1053/j.gastro.2004.07.006 15480987 10.1053/j.gastro.2004.07.006

[99] Stanke F, Ballmann M, Bronsveld I, Dörk T, Gallati S, Laabs U, Derichs N, Ritzka M, Posselt HG, Harms HK, Griese M, Blau H, Mastella G, Bijman J, Veeze H, Tümmler B (2008) Diversity of the basic defect of homozygous *CFTR* mutation genotypes in humans. J Med Genet 45:47–54. 10.1136/jmg.2007.053561 18178635 10.1136/jmg.2007.053561

[100] McClure ML, Barnes S, Brodsky JL, Sorscher EJ (2016) Trafficking and function of the cystic fibrosis transmembrane conductance regulator: a complex network of posttranslational modifications. Am J Physiol Lung Cell Mol Physiol 311:L719–L733. 10.1152/ajplung.00431.2015 27474090 10.1152/ajplung.00431.2015 PMC5142128

[101] Ward CL, Kopito RR (1994) Intracellular turnover of cystic fibrosis transmembrane conductance regulator. Inefficient processing and rapid degradation of wild-type and mutant proteins. J Biol Chem 269:25710–25718. 10.1016/S0021-9258(18)47306-1. 7523390

[102] Lukacs GL, Chang XB, Bear C, Kartner N, Mohamed A, Riordan JR, Grinstein S (1993) The delta F508 mutation decreases the stability of cystic fibrosis transmembrane conductance regulator in the plasma membrane. Determination of functional half-lives on transfected cells. J Biol Chem 268:21592–21598. 10.1016/S0021-9258(20)80582-1. 7691813

[103] van Barneveld A, Stanke F, Tamm S, Siebert B, Brandes G, Derichs N, Ballmann M, Junge S, Tümmler B (2010) Functional analysis of F508del CFTR in native human colon. Biochim Biophys Acta 1802:1062–1069. 10.1016/j.bbadis.2010.08.001 20696241 10.1016/j.bbadis.2010.08.001

[104] Dray-Charier N, Paul A, Scoazec JY, Veissière D, Mergey M, Capeau J, Soubrane O, Housset C (1999) Expression of delta F508 cystic fibrosis transmembrane conductance regulator protein and related chloride transport properties in the gallbladder epithelium from cystic fibrosis patients. Hepatology 29:1624–1634. 10.1002/hep.510290634 10347100 10.1002/hep.510290634

[105] Kälin N, Claass A, Sommer M, Puchelle E, Tümmler B (1999) DeltaF508 CFTR protein expression in tissues from patients with cystic fibrosis. J Clin Invest 103:1379–1389. 10.1172/JCI5731 10330420 10.1172/JCI5731 PMC408454

[106] Lancaster MA, Knoblich JA (2014) Organogenesis in a dish: modeling development and disease using organoid technologies. Science 345:1247125. 10.1126/science.1247125 25035496 10.1126/science.1247125

[107] Clevers H (2016) Modeling development and disease with organoids. Cell 165:1586–1597. 10.1016/j.cell.2016.05.082 27315476 10.1016/j.cell.2016.05.082

[108] Sato T, Stange DE, Ferrante M, Vries RG, Van Es JH, Van den Brink S, Van Houdt WJ, Pronk A, Van Gorp J, Siersema PD, Clevers H (2011) Long-term expansion of epithelial organoids from human colon, adenoma, adenocarcinoma, and Barrett’s epithelium. Gastroenterology 141:1762–1772. 10.1053/j.gastro.2011.07.050 21889923 10.1053/j.gastro.2011.07.050

[109] Dekkers JF, Berkers G, Kruisselbrink E, Vonk A, de Jonge HR, Janssens HM, Bronsveld I, van de Graaf EA, Nieuwenhuis EE, Houwen RH, Vleggaar FP, Escher JC, de Rijke YB, Majoor CJ, Heijerman HG, de Winter-de Groot KM, Clevers H, van der Ent CK, Beekman JM (2016) Characterizing responses to CFTR-modulating drugs using rectal organoids derived from subjects with cystic fibrosis. Sci Transl Med 8:344ra84. 10.1126/scitranslmed.aad8278. 10.1126/scitranslmed.aad8278 27334259

[110] Cuyx S, Ramalho AS, Fieuws S, Corthout N, Proesmans M, Boon M, Arnauts K, Carlon MS, Munck S, Dupont L, De Boeck K, Vermeulen F; Belgian Organoid Project (2024) Rectal organoid morphology analysis (ROMA) as a novel physiological assay for diagnostic classification in cystic fibrosis. Thorax 79:834-841. 10.1136/thorax-2023-220964 10.1136/thorax-2023-220964 39004507

[111] Sachs N, Papaspyropoulos A, Zomer-van Ommen DD, Heo I, Böttinger L, Klay D, Weeber F, Huelsz-Prince G, Iakobachvili N, Amatngalim GD, de Ligt J, van Hoeck A, Proost N, Viveen MC, Lyubimova A, Teeven L, Derakhshan S, Korving J, Begthel H, Dekkers JF, Kumawat K, Ramos E, van Oosterhout MF, Offerhaus GJ, Wiener DJ, Olimpio EP, Dijkstra KK, Smit EF, van der Linden M, Jaksani S, van de Ven M, Jonkers J, Rios AC, Voest EE, van Moorsel CH, van der Ent CK, Cuppen E, van Oudenaarden A, Coenjaerts FE, Meyaard L, Bont LJ, Peters PJ, Tans SJ, van Zon JS, Boj SF, Vries RG, Beekman JM, Clevers H (2019) Long-term expanding human airway organoids for disease modeling. EMBO J 38:e100300. 10.15252/embj.2018100300 30643021 10.15252/embj.2018100300 PMC6376275

[112] Schutgens F, Rookmaaker MB, Margaritis T, Rios A, Ammerlaan C, Jansen J, Gijzen L, Vormann M, Vonk A, Viveen M, Yengej FY, Derakhshan S, de Winter-de Groot KM, Artegiani B, van Boxtel R, Cuppen E, Hendrickx APA, van den Heuvel-Eibrink MM, Heitzer E, Lanz H, Beekman J, Murk JL, Masereeuw R, Holstege F, Drost J, Verhaar MC, Clevers H (2019) Tubuloids derived from human adult kidney and urine for personalized disease modeling. Nat Biotechnol 37:303–313. 10.1038/s41587-019-0048-8 30833775 10.1038/s41587-019-0048-8

[113] Berg P, Sorensen MV, Rousing AQ, Vebert Olesen H, Jensen-Fangel S, Jeppesen M, Leipziger J (2022) Challenged urine bicarbonate excretion as a measure of Cystic Fibrosis Transmembrane Conductance Regulator function in Cystic Fibrosis. Ann Intern Med 175:1543–1551. 10.7326/M22-1741 36315944 10.7326/M22-1741

[114] Rousing AQ, Jeppesen M, Jensen-Fangel S, Leipziger J, Sorensen MV, Berg P (2024) The challenged urine bicarbonate excretion test in cystic fibrosis: A comprehensive analysis of urine acid/base parameters. Acta Physiol (Oxf) 240:e14233. 10.1111/apha.14233 39308271 10.1111/apha.14233

[115] Wall SM, Verlander JW, Romero CA (2020) The renal physiology of pendrin-positive intercalated cells. Physiol Rev 100:1119–1147. 10.1152/physrev.00011.2019 32347156 10.1152/physrev.00011.2019 PMC7474261

[116] Berg P, Svendsen SL, Sorensen MV, Schreiber R, Kunzelmann K, Leipziger J (2021) The molecular mechanism of CFTR- and secretin-dependent renal bicarbonate excretion. J Physiol 599:3003–3011. 10.1113/JP281285 33963548 10.1113/JP281285

[117] Bae H, Kim BR, Jung S, Le J, van der Heide D, Yu W, Park SH, Hilkin BM, Gansemer ND, Powers LS, Kang T, Meyerholz DK, Schuster VL, Jang C, Welsh MJ (2024) Arteriovenous metabolomics in pigs reveals CFTR regulation of metabolism in multiple organs. J Clin Invest 134:e174500. 10.1172/JCI174500 38743489 10.1172/JCI174500 PMC11213515

[118] Molinski SV, Shahani VM, Subramanian AS, MacKinnon SS, Woollard G, Laforet M, Laselva O, Morayniss LD, Bear CE, Windemuth A (2018) Comprehensive mapping of cystic fibrosis mutations to CFTR protein identifies mutation clusters and molecular docking predicts corrector binding site. Proteins 86:833–843. 10.1002/prot.25496 29569753 10.1002/prot.25496

[119] Loo TW, Clarke DM (2017) Corrector VX-809 promotes interactions between cytoplasmic loop one and the first nucleotide-binding domain of CFTR. Biochem Pharmacol 136:24–31. 10.1016/j.bcp.2017.03.020 28366727 10.1016/j.bcp.2017.03.020

[120] Kleizen B, van Willigen M, Mijnders M, Peters F, Grudniewska M, Hillenaar T, Thomas A, Kooijman L, Peters KW, Frizzell R, van der Sluijs P, Braakman I (2021) Co-translational folding of the first transmembrane domain of ABC-transporter CFTR is supported by assembly with the first cytosolic domain. J Mol Biol 433:166955. 10.1016/j.jmb.2021.166955 33771570 10.1016/j.jmb.2021.166955

[121] Farinha CM, Sousa M, Canato S, Schmidt A, Uliyakina I, Amaral MD (2015) Increased efficacy of VX-809 in different cellular systems results from an early stabilization effect of F508del-CFTR. Pharmacol Res Perspect 3:e00152. 10.1002/prp2.152 26171232 10.1002/prp2.152 PMC4492728

[122] Farinha CM, King-Underwood J, Sousa M, Correia AR, Henriques BJ, Roxo-Rosa M, Da Paula AC, Williams J, Hirst S, Gomes CM, Amaral MD (2013) Revertants, low temperature, and correctors reveal the mechanism of F508del-CFTR rescue by VX-809 and suggest multiple agents for full correction. Chem Biol 20:943–955. 10.1016/j.chembiol.2013.06.004 23890012 10.1016/j.chembiol.2013.06.004

[123] He L, Kota P, Aleksandrov AA, Cui L, Jensen T, Dokholyan NV, Riordan JR (2013) Correctors of ΔF508 CFTR restore global conformational maturation without thermally stabilizing the mutant protein. FASEB J 27:536–545. 10.1096/fj.12-216119 23104983 10.1096/fj.12-216119 PMC3545534

[124] Loo TW, Bartlett MC, Clarke DM (2013) Corrector VX-809 stabilizes the first transmembrane domain of CFTR. Biochem Pharmacol 86:612–619. 10.1016/j.bcp.2013.06.028 23835419 10.1016/j.bcp.2013.06.028

[125] Ren HY, Grove DE, De La Rosa O, Houck SA, Sopha P, Van Goor F, Hoffman BJ, Cyr DM (2013) VX-809 corrects folding defects in cystic fibrosis transmembrane conductance regulator protein through action on membrane-spanning domain 1. Mol Biol Cell 24:3016–3024. 10.1091/mbc.E13-05-0240 23924900 10.1091/mbc.E13-05-0240 PMC3784376

[126] Hudson RP, Dawson JE, Chong PA, Yang Z, Millen L, Thomas PJ, Brouillette CG, Forman-Kay JD (2017) Direct binding of the corrector VX-809 to human CFTR NBD1: Evidence of an allosteric coupling between the binding site and the NBD1:CL4 interface. Mol Pharmacol 92:124–135. 10.1124/mol.117.108373 28546419 10.1124/mol.117.108373

[127] Laselva O, Molinski S, Casavola V, Bear CE (2018) Correctors of the major Cystic Fibrosis mutant interact through membrane-spanning domains. Mol Pharmacol 93:612–618. 10.1124/mol.118.111799 29618585 10.1124/mol.118.111799

[128] Krainer G, Schenkel M, Hartmann A, Ravamehr-Lake D, Deber CM, Schlierf M (2020) CFTR transmembrane segments are impaired in their conformational adaptability by a pathogenic loop mutation and dynamically stabilized by Lumacaftor. J Biol Chem 295:1985–1991. 10.1074/jbc.AC119.011360 31882543 10.1074/jbc.AC119.011360 PMC7029128

[129] Uluer AZ, MacGregor G, Azevedo P, Indihar V, Keating C, Mall MA, McKone EF, Ramsey BW, Rowe SM, Rubenstein RC, Taylor-Cousar JL, Tullis E, Yonker LM, Chu C, Lam AP, Nair N, Sosnay PR, Tian S, Van Goor F, Viswanathan L, Waltz D, Wang LT, Xi Y, Billings J, Horsley A; VX18-121-101; VX18-561-101 Study Groups (2023) Safety and efficacy of vanzacaftor-tezacaftor-deutivacaftor in adults with cystic fibrosis: randomised, double-blind, controlled, phase 2 trials. Lancet Respir Med 11:550-562. 10.1016/S2213-2600(22)00504-5 10.1016/S2213-2600(22)00504-5 PMC12815409 36842446

[130] Veit G, Roldan A, Hancock MA, Da Fonte DF, Xu H, Hussein M, Frenkiel S, Matouk E, Velkov T, Lukacs GL (2020) Allosteric folding correction of F508del and rare CFTR mutants by elexacaftor-tezacaftor-ivacaftor (Trikafta) combination. JCI Insight 5:e139983. 10.1172/jci.insight.139983 32853178 10.1172/jci.insight.139983 PMC7526550

[131] Becq F, Mirval S, Carrez T, Lévêque M, Billet A, Coraux C, Sage E, Cantereau A (2022) The rescue of F508del-CFTR by elexacaftor/tezacaftor/ivacaftor (Trikafta) in human airway epithelial cells is underestimated due to the presence of ivacaftor. Eur Respir J 59:2100671. 10.1183/13993003.00671-2021 34266939 10.1183/13993003.00671-2021

[132] Bongiorno R, Ludovico A, Moran O, Baroni D (2023) Elexacaftor mediates the rescue of F508del CFTR functional expression interacting with MSD2. Int J Mol Sci 24:12838. 10.3390/ijms241612838 37629017 10.3390/ijms241612838 PMC10454486

[133] Riepe C, Wąchalska M, Deol KK, Amaya AK, Porteus MH, Olzmann JA, Kopito RR (2024) Small-molecule correctors divert CFTR-F508del from ERAD by stabilizing sequential folding states. Mol Biol Cell 35:ar15. 10.1091/mbc.E23-08-0336. 10.1091/mbc.E23-08-0336 PMC10881158 38019608

[134] Ferreira FC, Buarque CD, Lopes-Pacheco M (2024) Organic synthesis and current understanding of the mechanisms of CFTR modulator drugs Ivacaftor, Tezacaftor, and Elexacaftor. Molecules 29:821. 10.3390/molecules29040821 38398574 10.3390/molecules29040821 PMC10891718

[135] Fiedorczuk K, Iordanov I, Mihályi C, Szollosi A, Csanády L, Chen J (2024) The structures of protein kinase A in complex with CFTR: Mechanisms of phosphorylation and noncatalytic activation. Proc Natl Acad Sci U S A 121:e2409049121. 10.1073/pnas.2409049121 39495916 10.1073/pnas.2409049121 PMC11573500

[136] Eckford PD, Li C, Ramjeesingh M, Bear CE (2012) Cystic fibrosis transmembrane conductance regulator (CFTR) potentiator VX-770 (ivacaftor) opens the defective channel gate of mutant CFTR in a phosphorylation-dependent but ATP-independent manner. J Biol Chem 287:36639–36649. 10.1074/jbc.M112.393637 22942289 10.1074/jbc.M112.393637 PMC3481266

[137] Jih KY, Hwang TC (2013) Vx-770 potentiates CFTR function by promoting decoupling between the gating cycle and ATP hydrolysis cycle. Proc Natl Acad Sci USA 110:4404–4409. 10.1073/pnas.1215982110 23440202 10.1073/pnas.1215982110 PMC3600496

[138] Csanády L, Töröcsik B (2019) Cystic fibrosis drug ivacaftor stimulates CFTR channels at picomolar concentrations. Elife 8:e46450. 10.7554/eLife.46450 31205003 10.7554/eLife.46450 PMC6594753

[139] Kopeikin Z, Yuksek Z, Yang HY, Bompadre SG (2014) Combined effects of VX-770 and VX-809 on several functional abnormalities of F508del-CFTR channels. J Cyst Fibros 13:508–514. 10.1016/j.jcf.2014.04.003 24796242 10.1016/j.jcf.2014.04.003

[140] Langron E, Prins S, Vergani P (2018) Potentiation of the cystic fibrosis transmembrane conductance regulator by VX-770 involves stabilization of the pre-hydrolytic O_1_ state. Br J Pharmacol 175:3990–4002. 10.1111/bph.14475 30107029 10.1111/bph.14475 PMC6151340

[141] Drumm ML, Wilkinson DJ, Smit LS, Worrell RT, Strong TV, Frizzell RA, Dawson DC, Collins FS (1991) Chloride conductance expressed by delta F508 and other mutant CFTRs in Xenopus oocytes. Science 254:1797–1799. 10.1126/science.1722350 1722350 10.1126/science.1722350

[142] Bompadre SG, Sohma Y, Li M, Hwang TC (2007) G551D and G1349D, two CF-associated mutations in the signature sequences of CFTR, exhibit distinct gating defects. J Gen Physiol 129:285–298. 10.1085/jgp.200609667 17353351 10.1085/jgp.200609667 PMC2151620

[143] Shaughnessy CA, Zeitlin PL, Bratcher PE (2021) Elexacaftor is a CFTR potentiator and acts synergistically with ivacaftor during acute and chronic treatment. Sci Rep 11:19810. 10.1038/s41598-021-99184-1 34615919 10.1038/s41598-021-99184-1 PMC8494914

[144] Veit G, Vaccarin C, Lukacs GL (2021) Elexacaftor co-potentiates the activity of F508del and gating mutants of CFTR. J Cyst Fibros 20:895–898. 10.1016/j.jcf.2021.03.011 33775603 10.1016/j.jcf.2021.03.011 PMC8463622

[145] Laselva O, Bartlett C, Gunawardena TNA, Ouyang H, Eckford PDW, Moraes TJ, Bear CE, Gonska T (2021) Rescue of multiple class II CFTR mutations by elexacaftor+tezacaftor+ivacaftor mediated in part by the dual activities of elexacaftor as both corrector and potentiator. Eur Respir J 57:2002774. 10.1183/13993003.02774-2020 33303536 10.1183/13993003.02774-2020 PMC8209484

[146] Fiedorczuk K, Chen J (2022) Molecular structures reveal synergistic rescue of Δ508 CFTR by Trikafta modulators. Science 378:284–290. 10.1126/science.ade2216 36264792 10.1126/science.ade2216 PMC9912939

[147] Collins FS (2019) Realizing the dream of molecularly targeted therapies for Cystic Fibrosis. N Engl J Med 381:1863–1865. 10.1056/NEJMe1911602 31670919 10.1056/NEJMe1911602

[148] Griese M, Costa S, Linnemann RW, Mall MA, McKone EF, Polineni D, Quon BS, Ringshausen FC, Taylor-Cousar JL, Withers NJ, Moskowitz SM, Daines CL (2021) Safety and efficacy of Elexacaftor/Tezacaftor/Ivacaftor for 24 weeks or longer in people with Cystic Fibrosis and one or more F508del alleles: Interim results of an open-label phase 3 clinical trial. Am J Respir Crit Care Med 203:381–385. 10.1164/rccm.202008-3176LE 32969708 10.1164/rccm.202008-3176LE PMC8020728

[149] Daines CL, Tullis E, Costa S, Linnemann RW, Mall MA, McKone EF, Polineni D, Quon BS, Ringshausen FC, Rowe SM, Selvadurai H, Taylor-Cousar JL, Withers NJ, Ahluwalia N, Moskowitz SM, Prieto-Centurion V, Tan YV, Tian S, Weinstock T, Xuan F, Zhang Y, Ramsey B, Griese M; VX17-445-105 Study Group (2023) Long-term safety and efficacy of elexacaftor/tezacaftor/ivacaftor in people with cystic fibrosis and at least one F508del allele: 144-week interim results from a 192-week open-label extension study. Eur Respir J 62: 2202029. 10.1183/13993003.02029-2022 10.1183/13993003.02029-2022 PMC10701091 37945033

[150] Zemanick ET, Taylor-Cousar JL, Davies J, Gibson RL, Mall MA, McKone EF, McNally P, Ramsey BW, Rayment JH, Rowe SM, Tullis E, Ahluwalia N, Chu C, Ho T, Moskowitz SM, Noel S, Tian S, Waltz D, Weinstock TG, Xuan F, Wainwright CE, McColley SA (2021) A phase 3 open-label study of Elexacaftor/Tezacaftor/Ivacaftor in children 6 through 11 years of age with Cystic Fibrosis and at least one F508del allele. Am J Respir Crit Care Med 203:1522–1532. 10.1164/rccm.202102-0509OC 33734030 10.1164/rccm.202102-0509OC PMC8483230

[151] Barry PJ, Mall MA, Álvarez A, Colombo C, de Winter-de Groot KM, Fajac I, McBennett KA, McKone EF, Ramsey BW, Sutharsan S, Taylor-Cousar JL, Tullis E, Ahluwalia N, Jun LS, Moskowitz SM, Prieto-Centurion V, Tian S, Waltz D, Xuan F, Zhang Y, Rowe SM, Polineni D; VX18-445-104 Study Group (2021) Triple therapy for Cystic Fibrosis Phe508del-gating and -residual function genotypes. N Engl J Med 385:815–825. 10.1056/NEJMoa2100665 10.1056/NEJMoa2100665 PMC8982185 34437784

[152] Wainwright C, McColley SA, McNally P, Powers M, Ratjen F, Rayment JH, Retsch-Bogart G, Roesch E, Ahluwalia N, Chin A, Chu C, Lu M, Menon P, Waltz D, Weinstock T, Zelazoski L, Davies JC (2023) Long-term safety and efficacy of Elexacaftor/Tezacaftor/Ivacaftor in children aged ⩾6 years with Cystic Fibrosis and at least one F508del allele: A phase 3, open-label clinical trial. Am J Respir Crit Care Med 208:68–78. 10.1164/rccm.202301-0021OC 37154609 10.1164/rccm.202301-0021OC PMC10870850

[153] Bower JK, Volkova N, Ahluwalia N, Sahota G, Xuan F, Chin A, Weinstock TG, Ostrenga J, Elbert A (2023) Real-world safety and effectiveness of elexacaftor/tezacaftor/ivacaftor in people with cystic fibrosis: Interim results of a long-term registry-based study. J Cyst Fibros 22:730–737. 10.1016/j.jcf.2023.03.002 36963986 10.1016/j.jcf.2023.03.002

[154] Leo-Hansen C, Faurholt-Jepsen D, Qvist T, Højte C, Nielsen BU, Bryrup T, Henriksen EH, Katzenstein T, Skov M, Mathiesen IHM, Jeppesen M, Jensen-Fangel S, Olesen HV, Buchvald FF, Nielsen KG, Jimenez-Solem E, Ritz C, Pressler T, Olsen MF; TransformCF Study Group (2024) Lung function improvement on triple modulators: high-resolution, nationwide data from the Danish Cystic Fibrosis Cohort. ERJ Open Res 10:00339-2024. 10.1183/23120541.00339-2024 10.1183/23120541.00339-2024 PMC11626609 39655171

[155] Bryrup T, Faurholt-Jepsen D, Pressler T, Henriksen EH, Leo-Hansen C, Nielsen BU, Højte C, Mathiesen IHM, Katzenstein TL, Jeppesen M, Jensen-Fangel S, Olesen HV, Skov M, Qvist T, Olsen MF; TransformCF Study Group (2024) Real-world data confirm elexacftor/tezacaftor/ivacaftor modulators halves sweat chloride concentration in eligible people with cystic fibrosis. APMIS 132:728-733. 10.1111/apm.13453 10.1111/apm.13453 39092470

[156] Fragoso E, Boaventura R, Almeida L, Amorim A, Gamboa F, Santos AS, Gonçalves F, Cruz CM, Carreiro A, Gonçalves AS, Teixeira V, Azevedo P (2024) Elexacaftor/tezacaftor/ivacaftor, a game-changer in cystic fibrosis: The Portuguese experience. Pulm Pharmacol Ther 87:102328. 10.1016/j.pupt.2024.102328 39299648 10.1016/j.pupt.2024.102328

[157] Graeber SY, Vitzthum C, Pallenberg ST, Naehrlich L, Stahl M, Rohrbach A, Drescher M, Minso R, Ringshausen FC, Rueckes-Nilges C, Klajda J, Berges J, Yu Y, Scheuermann H, Hirtz S, Sommerburg O, Dittrich AM, Tümmler B, Mall MA (2022) Effects of Elexacaftor/Tezacaftor/Ivacaftor therapy on CFTR Function in patients with Cystic Fibrosis and one or two F508del alleles. Am J Respir Crit Care Med 205:540–549. 10.1164/rccm.202110-2249OC 34936849 10.1164/rccm.202110-2249OC

[158] Keens T, Hoffman V, Topuria I, Elder K, Cerf S, Mulder K, Roberts J, Lysinger J, Del Carmen Reyes M, Berdella M, Cairns AM, Jain M, Ganapathy V, Lou Y, Morcos B, Wu C, Sass L; VX19-CFD-003 Study Group (2024) Real-world effectiveness of elexacaftor/tezacaftor/ivacaftor on the burden of illness in adolescents and adults with cystic fibrosis. Heliyon 10:e28508. 10.1016/j.heliyon.2024.e28508 10.1016/j.heliyon.2024.e28508 PMC10998118 38586424

[159] Salomão LZ, Athanazio RA, Rached SZ, Lopes-Pacheco M, Camargo M (2023) A real-life study of elexacaftor-tezacaftor-ivacaftor therapy in people with cystic fibrosis in Brazil. Pulmonology 29:543–545. 10.1016/j.pulmoe.2023.03.008 37210338 10.1016/j.pulmoe.2023.03.008

[160] Olivier M, Kavvalou A, Welsner M, Hirtz R, Straßburg S, Sutharsan S, Stehling F, Steindor M (2023) Real-life impact of highly effective CFTR modulator therapy in children with cystic fibrosis. Front Pharmacol 14:1176815. 10.3389/fphar.2023.1176815 37229253 10.3389/fphar.2023.1176815 PMC10203630

[161] Schütz K, Pallenberg ST, Kontsendorn J, DeLuca D, Sukdolak C, Minso R, Büttner T, Wetzke M, Dopfer C, Sauer-Heilborn A, Ringshausen FC, Junge S, Tümmler B, Hansen G, Dittrich AM (2023) Spirometric and anthropometric improvements in response to elexacaftor/tezacaftor/ivacaftor depending on age and lung disease severity. Front Pharmacol 14:1171544. 10.3389/fphar.2023.1171544 37469865 10.3389/fphar.2023.1171544 PMC10352657

[162] Daccò V, Rosazza C, Mariani A, Rizza C, Ingianni N, Nazzari E, Terlizzi V, Blasi FA, Alicandro G (2024) Effectiveness and safety of elexacaftor/tezacaftor/ivacaftor treatment in children aged 6–11 years with cystic fibrosis in a real-world setting. Pediatr Pulmonol 59:2792–2799. 10.1002/ppul.27125 38869349 10.1002/ppul.27125

[163] Alicandro G, Gramegna A, Bellino F, Sciarrabba SC, Lanfranchi C, Contarini M, Retucci M, Daccò V, Blasi F (2024) Heterogeneity in response to Elexacaftor/Tezacaftor/Ivacaftor in people with cystic fibrosis. J Cyst Fibros 23:1072–1079. 10.1016/j.jcf.2024.04.013 38729849 10.1016/j.jcf.2024.04.013

[164] Savi D, Lucca F, Tridello G, Meneghelli I, Comello I, Tomezzoli S, Signorini M, Proietti E, Cucchetto G, Volpi S, Cipolli M (2023) Long-term clinical outcomes of elexacaftor/tezacaftor/ivacaftor therapy in adults with cystic fibrosis and advanced pulmonary disease. Respir Med 219:107406. 10.1016/j.rmed.2023.107406 37690570 10.1016/j.rmed.2023.107406

[165] Kos R, Neerincx AH, Fenn DW, Brinkman P, Lub R, Vonk SEM, Roukema J, Reijers MH, Terheggen-Lagro SWJ, Altenburg J, Majoor CJ, Bos LD, Haarman EG, Maitland-van der Zee AH; Amsterdam Mucociliary Clearance Disease (AMCD) Research Group (2022) Real-life efficacy and safety of elexacaftor/tezacaftor/ivacaftor on severe cystic fibrosis lung disease patients. Pharmacol Res Perspect 10:e01015. 10.1002/prp2.1015 10.1002/prp2.1015 PMC9703582 36440690

[166] Lopes K, Custódio C, Lopes C, Bolas R, Azevedo P (2023) Elexacaftor/tezacaftor/ivacaftor-real-world clinical effectiveness and safety. A single-center Portuguese study. J Bras Pneumol 49:e20220312. 10.36416/1806-3756/e20220312. 10.36416/1806-3756/e20220312 PMC9970613 36820745

[167] Gj C, Maguire S, Tc L, Scanlan L, Ss S, Muthukumarana T, Bevan A, Keogh Rh, Jp L (2024) Real-world impact of Elexacaftor-Tezacaftor-Ivacaftor treatment in young people with Cystic Fibrosis: A longitudinal study. Respir Med 236:107882. 10.1016/j.rmed.2024.107882 39581272 10.1016/j.rmed.2024.107882

[168] Urquhart DS, Dowle H, Moffat K, Forster J, Cunningham S, Macleod KA (2024) Lung clearance index (LCI2,5) changes after initiation of Elexacaftor/Tezacaftor/Ivacaftor in children with cystic fibrosis aged between 6 and 11 years: The “real-world” differs from trial data. Pediatr Pulmonol 59:1449–1453. 10.1002/ppul.26938 38415920 10.1002/ppul.26938

[169] Proud D, Duckers J (2023) Weight a minute: Exploring the effect on weight and body composition after the initiation of elexacaftor/tezacaftor/ivacaftor in adults with CF. J Cyst Fibros 22:847–850. 10.1016/j.jcf.2023.06.002 37355345 10.1016/j.jcf.2023.06.002

[170] Vijaykumar K, Leung HM, Barrios A, Wade J, Hathorne HY, Nichols DP, Tearney GJ, Rowe SM, Solomon GM (2024) Longitudinal improvements in clinical and functional outcomes following initiation of elexacaftor/tezacaftor/ivacaftor in patients with cystic fibrosis. Heliyon 10:e29188. 10.1016/j.heliyon.2024.e29188 38681615 10.1016/j.heliyon.2024.e29188 PMC11052906

[171] Graeber SY, Renz DM, Stahl M, Pallenberg ST, Sommerburg O, Naehrlich L, Berges J, Dohna M, Ringshausen FC, Doellinger F, Vitzthum C, Röhmel J, Allomba C, Hämmerling S, Barth S, Rückes-Nilges C, Wielpütz MO, Hansen G, Vogel-Claussen J, Tümmler B, Mall MA, Dittrich AM (2022) Effects of Elexacaftor/Tezacaftor/Ivacaftor therapy on lung clearance index and magnetic resonance imaging in patients with Cystic Fibrosis and one or two F508del alleles. Am J Respir Crit Care Med 206:311–320. 10.1164/rccm.202201-0219OC 35536314 10.1164/rccm.202201-0219OC

[172] Streibel C, Willers CC, Pusterla O, Bauman G, Stranzinger E, Brabandt B, Bieri O, Curdy M, Bullo M, Frauchiger BS, Korten I, Krüger L, Casaulta C, Ratjen F, Latzin P, Kieninger E (2023) Effects of elexacaftor/tezacaftor/ivacaftor therapy in children with cystic fibrosis - a comprehensive assessment using lung clearance index, spirometry, and functional and structural lung MRI. J Cyst Fibros 22:615–622. 10.1016/j.jcf.2022.12.012 36635199 10.1016/j.jcf.2022.12.012

[173] Appelt D, Steinkamp G, Sieber S, Ellemunter H (2023) Early and sustained improvements of lung clearance index from two to sixteen weeks of elexacaftor/tezacaftor/ivacaftor therapy in patients with cystic fibrosis-a real world study. Front Pharmacol 14:1125853. 10.3389/fphar.2023.1125853 36969845 10.3389/fphar.2023.1125853 PMC10030732

[174] Fainardi V, Skenderaj K, Ciuni A, Milanese G, Deolmi M, Longo F, Spaggiari C, Sverzellati N, Esposito S, Pisi G (2023) Structural changes in lung morphology detected by MRI after modulating therapy with elexacaftor/tezacaftor/ivacaftor in adolescent and adult patients with cystic fibrosis. Respir Med 216:107328. 10.1016/j.rmed.2023.107328 37321310 10.1016/j.rmed.2023.107328

[175] McNally P, Lester K, Stone G, Elnazir B, Williamson M, Cox D, Linnane B, Kirwan L, Rea D, O'Regan P, Semple T, Saunders C, Tiddens HAWM, McKone E, Davies JC; RECOVER Study Group (2023) Improvement in lung clearance index and chest computed tomography scores with Elexacaftor/Tezacaftor/Ivacaftor treatment in people with Cystic Fibrosis aged 12 years and older - The RECOVER trial. Am J Respir Crit Care Med 208:917-929. 10.1164/rccm.202308-1317OC 10.1164/rccm.202308-1317OC 37703083

[176] Klimeš F, Voskrebenzev A, Gutberlet M, Speth M, Grimm R, Dohna M, Hansen G, Wacker F, Renz DM, Dittrich AM, Vogel-Claussen J (2024) Effect of CFTR modulator therapy with elexacaftor/tezacaftor/ivacaftor on pulmonary ventilation derived by 3D phase-resolved functional lung MRI in cystic fibrosis patients. Eur Radiol 34:80–89. 10.1007/s00330-023-09912-6 37548691 10.1007/s00330-023-09912-6 PMC10791851

[177] David M, Benlala I, Bui S, Benkert T, Berger P, Laurent F, Macey J, Dournes G (2024) Longitudinal evaluation of bronchial changes in Cystic Fibrosis patients undergoing Elexacaftor/Tezacaftor/Ivacaftor therapy using lung MRI with ultrashort echo-times. J Magn Reson Imaging 60:116–124. 10.1002/jmri.29041 37861357 10.1002/jmri.29041

[178] Dohna M, Voskrebenzev A, Klimeš F, Kaireit TF, Glandorf J, Pallenberg ST, Ringshausen FC, Hansen G, Renz DM, Wacker F, Dittrich AM, Vogel-Claussen J (2024) PREFUL MRI for monitoring perfusion and ventilation changes after Elexacaftor-Tezacaftor-Ivacaftor therapy for Cystic Fibrosis: A feasibility study. Radiol Cardiothorac Imaging 6:e230104. 10.1148/ryct.230104 38573129 10.1148/ryct.230104 PMC11056757

[179] Stahl M, Dohna M, Graeber SY, Sommerburg O, Renz DM, Pallenberg ST, Voskrebenzev A, Schütz K, Hansen G, Doellinger F, Steinke E, Thee S, Röhmel J, Barth S, Rückes-Nilges C, Berges J, Hämmerling S, Wielpütz MO, Naehrlich L, Vogel-Claussen J, Tümmler B, Mall MA, Dittrich AM (2024) Impact of elexacaftor/tezacaftor/ivacaftor therapy on lung clearance index and magnetic resonance imaging in children with cystic fibrosis and one or two F508del alleles. Eur Respir J 64:2400004. 10.1183/13993003.00004-2024 38901883 10.1183/13993003.00004-2024 PMC11375515

[180] Alam F, Munidasa S, Zanette B, Braganza S, Li D, Jensen R, Dumas MP, Ratjen F, Santyr G (2024) Assessing ^129^Xe multi-breath washout MRI response to elexacaftor/tezacaftor/ivacaftor intervention in pediatric CF. J Cyst Fibros 30:S1569–1993(24)01789–2. 10.1016/j.jcf.2024.09.021. 10.1016/j.jcf.2024.09.021 39353741

[181] Daccò V, Gramegna A, Rosazza C, Mariani A, Biffi A, Lanfranchi C, Zazzeron L, Bellante F, Blasi F, Alicandro G (2024) Lung clearance index improves in people with Cystic Fibrosis not achieving a clinical important difference in forced expiratory volume in one second after Elexacaftor/Tezacaftor/ Ivacaftor therapy. Lung 203:9. 10.1007/s00408-024-00768-1 39614886 10.1007/s00408-024-00768-1

[182] Cazier P, Chassagnon G, Dhote T, Da Silva J, Kanaan R, Honore I, Carlier N, Revel MP, Canniff E, Martin C, Burgel PR (2024) Reversal of cylindrical bronchial dilatations in a subset of adults with cystic fibrosis treated with elexacaftor-tezacaftor-ivacaftor. Eur Respir J 8:2301794. 10.1183/13993003.01794-2023 10.1183/13993003.01794-2023 38331460

[183] Wucherpfennig L, Triphan SMF, Wege S, Kauczor HU, Heussel CP, Sommerburg O, Stahl M, Mall MA, Eichinger M, Wielpütz MO (2023) Elexacaftor/Tezacaftor/Ivacaftor improves bronchial artery dilatation detected by magnetic resonance imaging in patients with Cystic Fibrosis. Ann Am Thorac Soc 20:1595–1604. 10.1513/AnnalsATS.202302-168OC 37579262 10.1513/AnnalsATS.202302-168OC

[184] Jones JT, Morelli KA, Vesely EM, Puerner CTS, Pavuluri CK, Ross BS, van Rhijn N, Bromley MJ, Cramer RA (2023). The cystic fibrosis treatment Trikafta affects the growth, viability, and cell wall of *Aspergillus fumigatus* biofilms. mBio 14:e0151623. 10.1128/mbio.01516-23. 10.1128/mbio.01516-23 PMC10653927 37830825

[185] Morgan SJ, Nichols DP, Ni W, Hong G, Salipante SJ, Solomon GM, Rowe SM, Clancy JP, Cramer RA, Singh PK (2024) Elexacaftor/Tezacaftor/Ivacaftor markedly reduces *Aspergillus fumigatus* in Cystic Fibrosis. Am J Respir Crit Care Med 210:1155–1158. 10.1164/rccm.202406-1128RL 39189854 10.1164/rccm.202406-1128RL PMC12042367

[186] Dittrich AM, Sieber S, Naehrlich L, Burkhart M, Hafkemeyer S, Tümmler B; Registry Working Group of the German CF Registry (2024) Use of elexacaftor/tezacaftor/ivacaftor leads to changes in detection frequencies of *Staphylococcus aureus* and *Pseudomonas aeruginosa* dependent on age and lung function in people with cystic fibrosis. Int J Infect Dis 139:124-131. 10.1016/j.ijid.2023.11.013 10.1016/j.ijid.2023.11.013 38036261

[187] Armbruster CR, Hilliam YK, Zemke AC, Atteih S, Marshall CW, Moore J, Koirala J, Krainz L, Gaston JR, Lee SE, Cooper VS, Bomberger JM (2024) Persistence and evolution of *Pseudomonas aeruginosa* following initiation of highly effective modulator therapy in cystic fibrosis. mBio 15:e0051924. 10.1128/mbio.00519-24. 10.1128/mbio.00519-24 PMC11077959 38564694

[188] Ledger EL, Smith DJ, Teh JJ, Wood ME, Whibley PE, Morrison M, Goldberg JB, Reid DW, Wells TJ (2024) Impact of CFTR modulation on *Pseudomonas aeruginosa* infection in people with Cystic Fibrosis. J Infect Dis 230:e536–e547. 10.1093/infdis/jiae051 38442240 10.1093/infdis/jiae051 PMC11420785

[189] McParland C, Nunn M, Marras TK, Chiasson M (2024) Eradication of *Mycobacterium abscessus* infection in cystic fibrosis with initiation of Elexacaftor/Tezacaftor/Ivacaftor. J Cyst Fibros 23:38–40. 10.1016/j.jcf.2023.03.021 37076409 10.1016/j.jcf.2023.03.021

[190] Wiesel V, Aviram M, Mei-Zahav M, Dotan M, Prais D, Cohen-Cymberknoh M, Gur M, Bar-Yoseph R, Livnat G, Goldbart A, Hazan G, Hazan I, Golan-Tripto I (2024) Eradication of nontuberculous mycobacteria in people with Cystic Fibrosis treated with Elexacaftor/Tezacaftor/Ivacaftor: A multicenter cohort study. J Cyst Fibros 23:41–49. 10.1016/j.jcf.2023.05.003 37173154 10.1016/j.jcf.2023.05.003

[191] Gavey R, Nolan J, Moore V, Reid D, Brown J (2024) Clinical and radiological improvement of cavitary *Mycobacteroides abscessus* disease in cystic fibrosis following initiation of elexacaftor/tezacaftor/ivacaftor. J Cyst Fibros 23:1024–1026. 10.1016/j.jcf.2024.05.008 38777631 10.1016/j.jcf.2024.05.008

[192] Nichols DP, Morgan SJ, Skalland M, Vo AT, Van Dalfsen JM, Singh SB, Ni W, Hoffman LR, McGeer K, Heltshe SL, Clancy JP, Rowe SM, Jorth P, Singh PK; PROMISE-Micro Study Group (2023) Pharmacologic improvement of CFTR function rapidly decreases sputum pathogen density, but lung infections generally persist. J Clin Invest 133:e167957. 10.1172/JCI167957 10.1172/JCI167957 PMC10178839 36976651

[193] Morgan SJ, Coulter E, Betts HL, Solomon GM, Clancy JP, Rowe SM, Nichols DP, Singh PK; PROMISE-Micro Study Group (2024) Elexacaftor/tezacaftor/ivacaftor's effects on cystic fibrosis infections are maintained, but not increased, after 3.5 years of treatment. J Clin Invest 134:e184171. 10.1172/JCI184171. 10.1172/JCI184171 PMC11473140 39235967

[194] Mianowski L, Doléans-Jordheim A, Barraud L, Rabilloud M, Richard M, Josserand RN, Durieu I, Reynaud Q (2024) One year of ETI reduces lung bacterial colonisation in adults with cystic fibrosis. Sci Rep 14:29298. 10.1038/s41598-024-77246-4 39592637 10.1038/s41598-024-77246-4 PMC11599715

[195] Sosinski LM, H CM, Neugebauer KA, Ghuneim LJ, Guzior DV, Castillo-Bahena A, Mielke J, Thomas R, McClelland M, Conrad D, Quinn RA (2022) A restructuring of microbiome niche space is associated with Elexacaftor-Tezacaftor-Ivacaftor therapy in the cystic fibrosis lung. J Cyst Fibros 21:996-1005. 10.1016/j.jcf.2021.11.003 10.1016/j.jcf.2021.11.003 PMC9124239 34824018

[196] Pallenberg ST, Pust MM, Rosenboom I, Hansen G, Wiehlmann L, Dittrich AM, Tümmler B (2022) Impact of Elexacaftor/Tezacaftor/Ivacaftor therapy on the Cystic Fibrosis airway microbial metagenome. Microbiol Spectr 10:e0145422. 10.1128/spectrum.01454-22 36154176 10.1128/spectrum.01454-22 PMC9602284

[197] Martin C, Guzior DV, Gonzalez CT, Okros M, Mielke J, Padillo L, Querido G, Gil M, Thomas R, McClelland M, Conrad D, Widder S, Quinn RA (2023) Longitudinal microbial and molecular dynamics in the cystic fibrosis lung after Elexacaftor-Tezacaftor-Ivacaftor therapy. Respir Res 24:317. 10.1186/s12931-023-02630-z 38104128 10.1186/s12931-023-02630-z PMC10725582

[198] Yule A, Ng C, Recto A, Lockwood F, Dellschaft NS, Hoad CL, Zagoya C, Mainz JG, Major G, Barr HL, Gowland PA, Stewart I, Marciani L, Spiller RC, Smyth AR (2024) A longitudinal study assessing the impact of elexacaftor/tezacaftor/ivacaftor on gut transit and function in people with cystic fibrosis using magnetic resonance imaging (MRI). J Cyst Fibros 23:984–990. 10.1016/j.jcf.2024.08.001 39242338 10.1016/j.jcf.2024.08.001

[199] Calthorpe RJ, Goodchild N, Gleetus V, Premakumar V, Hayee B, Elliott Z, Evans B, Rowbotham NJ, Carr SB, Barr H, Horsley A, Peckham D, Smyth AR (2024) A grumbling concern: A survey of gastrointestinal symptoms in cystic fibrosis in the modulator era. NIHR Open Res 3:18. 10.3310/nihropenres.13384.1 37881465 10.3310/nihropenres.13384.2 PMC10593346

[200] Schwarzenberg SJ, Vu PT, Skalland M, Hoffman LR, Pope C, Gelfond D, Narkewicz MR, Nichols DP, Heltshe SL, Donaldson SH, Frederick CA, Kelly A, Pittman JE, Ratjen F, Rosenfeld M, Sagel SD, Solomon GM, Stalvey MS, Clancy JP, Rowe SM, Freedman SD; Promise Study Group (2023) Elexacaftor/tezacaftor/ivacaftor and gastrointestinal outcomes in cystic fibrosis: Report of promise-GI. J Cyst Fibros 22:282-289. 10.1016/j.jcf.2022.10.003 10.1016/j.jcf.2022.10.003 PMC10144072 36280527

[201] Mainz JG, Lester K, Elnazir B, Williamson M, McKone E, Cox D, Linnane B, Zagoya C, Duckstein F, Barucha A, Davies JC, McNally P; RECOVER Study Group (2024) Reduction in abdominal symptoms (CFAbd-Score), faecal M2-pyruvate-kinase and Calprotectin over one year of treatment with Elexacaftor-Tezacaftor-Ivacaftor in people with CF aged ≥12 years - The RECOVER study. J Cyst Fibros 23:474-480. 10.1016/j.jcf.2023.10.001 10.1016/j.jcf.2023.10.001 37806792

[202] Stastna N, Kunovsky L, Svoboda M, Pokojova E, Homola L, Mala M, Gracova Z, Jerabkova B, Skrickova J, Trna J (2024) Improved nutritional outcomes and gastrointestinal symptoms in adult Cystic Fibrosis patients treated with Elexacaftor/Tezacaftor/Ivacaftor. Dig Dis 42:361–368. 10.1159/000538606 38569478 10.1159/000538606 PMC11250406

[203] Mouzaki M, Dupuis A, Avolio J, Griffin K, Ratjen F, Tullis E, Gonska T (2023) Weight increase in people with cystic fibrosis on CFTR modulator therapy is mainly due to increase in fat mass. Front Pharmacol 14:157459. 10.3389/fphar.2023.1157459 10.3389/fphar.2023.1157459 PMC10372433 37521467

[204] Merino Sánchez-Cañete A, López Cárdenes CM, Vicente Santamaría S, Gutiérrez Martínez JR, Suárez González M, Álvarez Merino M, González Jiménez D (2024) Increased fat mass and obesity risk after elexacaftor-tezacaftor-ivacaftor therapy in young adults with cystic fibrosis. Front Nutr 11:1477674. 10.3389/fnut.2024.1477674 39582664 10.3389/fnut.2024.1477674 PMC11582979

[205] Ratti GA, Smith H, Mirfakhraee S, Reisch J, Cohen L, Jain R, Finklea JD (2025) Development of metabolic syndrome in people with Cystic Fibrosis one year after exposure to elexacaftor-tezacaftor-ivacaftor. J Cyst Fibros 24:47–52. 10.1016/j.jcf.2024.09.022 39419654 10.1016/j.jcf.2024.09.022

[206] Goralski JL, Hoppe JE, Mall MA, McColley SA, McKone E, Ramsey B, Rayment JH, Robinson P, Stehling F, Taylor-Cousar JL, Tullis E, Ahluwalia N, Chin A, Chu C, Lu M, Niu T, Weinstock T, Ratjen F, Rosenfeld M (2023) Phase 3 open-label clinical trial of Elexacaftor/Tezacaftor/Ivacaftor in children aged 2–5 years with Cystic Fibrosis and at least one F508del allele. Am J Respir Crit Care Med 208:59–67. 10.1164/rccm.202301-0084OC 36921081 10.1164/rccm.202301-0084OC PMC10870849

[207] Zemanick ET, Ramsey B, Sands D, McKone EF, Fajac I, Taylor-Cousar JL, Mall MA, Konstan MW, Nair N, Zhu J, Arteaga-Solis E, Van Goor F, McGarry L, Prieto-Centurion V, Sosnay PR, Bozic C, Waltz D, Mayer-Hamblett N (2025) Sweat chloride reflects CFTR function and correlates with clinical outcomes following CFTR modulator treatment. J Cyst Fibros 24:246–254. 10.1016/j.jcf.2024.12.006 39755444 10.1016/j.jcf.2024.12.006

[208] Zemanick ET, Emerman I, McCreary M, Mayer-Hamblett N, Warden MN, Odem-Davis K, VanDevanter DR, Ren CL, Young J, Konstan MW; CHEC-SC Study Group (2024) Heterogeneity of CFTR modulator-induced sweat chloride concentrations in people with cystic fibrosis. J Cyst Fibros 23:676-684. 10.1016/j.jcf.2024.02.001 10.1016/j.jcf.2024.02.001 PMC11322419 38360461

[209] Boëlle PY, Viviani L, Busson PF, Olesen HV, Ravilly S, Stern M, Assael BM, Barreto C, Drevinek P, Thomas M, Krivec U, Mei-Zahav M, Vibert JF, Clement A, Mehta A, Corvol H, French CF Modifier Gene Study Investigators, European CF Registry Working Group (2012) Reference percentiles for FEV(1) and BMI in European children and adults with cystic fibrosis. Orphanet J Rare Dis 7:64. 10.1186/1750-1172-7-64 10.1186/1750-1172-7-64 PMC3520808 22958330

[210] Dittrich AS, Dumke M, Kapl F, Schneider P, Wege S, Gräber S, Stahl M, Herth FJ, Naehrlich L, Mall MA, Sommerburg O, Registry GCF (2023) Survival-adjusted FEV1 and BMI percentiles for patients with Cystic Fibrosis before the era of triple CFTR modulator therapy in Germany. Respiration 102:1. 10.1159/000529524 37062281 10.1159/000529524

[211] Pienkowska K, Pust MM, Gessner M, Gaedcke S, Thavarasa A, Rosenboom I, Morán Losada P, Minso R, Arnold C, Hedtfeld S, Dorda M, Wiehlmann L, Mainz JG, Klockgether J, Tümmler B (2023) The Cystic Fibrosis upper and lower airway metagenome. Microbiol Spectr 11:e0363322. 10.1128/spectrum.03633-22 36892308 10.1128/spectrum.03633-22 PMC10101124

[212] Stanke F, Pallenberg ST, Tamm S, Hedtfeld S, Eichhorn EM, Minso R, Hansen G, Welte T, Sauer-Heilborn A, Ringshausen FC, Junge S, Tümmler B, Dittrich AM (2023) Changes in cystic fibrosis transmembrane conductance regulator protein expression prior to and during elexacaftor-tezacaftor-ivacaftor therapy. Front Pharmacol 14:1114584. 10.3389/fphar.2023.1114584 36778025 10.3389/fphar.2023.1114584 PMC9911415

[213] Cinek O, Furstova E, Novotna S, Hubackova K, Dousova T, Borek-Dohalska L, Drevinek P (2025) Gene expression profile of intestinal organoids from people with cystic fibrosis upon exposure to elexacaftor/tezacaftor/ivacaftor. J Cyst Fibros 24:157–163. 10.1016/j.jcf.2024.09.005 39278758 10.1016/j.jcf.2024.09.005

[214] Yue M, Weiner DJ, Gaietto KM, Rosser FJ, Qoyawayma CM, Manni ML, Myerburg MM, Pilewski JM, Celedón JC, Chen W, Forno E (2024) Nasal epithelium transcriptomics predict clinical response to Elexacaftor/Tezacaftor/Ivacaftor. Am J Respir Cell Mol Biol 71:730–739. 10.1165/rcmb.2024-0103OC 39028582 10.1165/rcmb.2024-0103OC PMC11622631

[215] Bihler H, Sivachenko A, Millen L, Bhatt P, Patel AT, Chin J, Bailey V, Musisi I, LaPan A, Allaire NE, Conte J, Simon NR, Magaret AS, Raraigh KS, Cutting GR, Skach WR, Bridges RJ, Thomas PJ, Mense M (2024) In vitro modulator responsiveness of 655 CFTR variants found in people with cystic fibrosis. J Cyst Fibros 23:664–675. 10.1016/j.jcf.2024.02.006 38388235 10.1016/j.jcf.2024.02.006

[216] Cromwell EA, Ostrenga JS, Sanders DB, Morgan W, Castellani C, Szczesniak R, Burgel PR (2024) Impact of the expanded label for elexacaftor/tezacaftor/ivacaftor in people with cystic fibrosis with no F508del variant in the USA. Eur Respir J 64:2401146. 10.1183/13993003.01146-2024 39227072 10.1183/13993003.01146-2024 PMC11561404

[217] Kroes S, Bierlaagh MC, Lefferts JW, Boni A, Muilwijk D, Viscomi C, Keijzer-Nieuwenhuijze NDA, Cristiani L, Niemöller PJ, Verburg TF, Cutrera R, Fiocchi AG, Lucidi V, van der Ent CK, Beekman JM, Alghisi F, Ciciriello F (2025) Elexacaftor/tezacaftor/ivacaftor efficacy in intestinal organoids with rare CFTR variants in comparison to CFTR-F508del and CFTR-wild type controls. J Cyst Fibros 24:175–182. 10.1016/j.jcf.2024.09.019 39523185 10.1016/j.jcf.2024.09.019

[218] Lefferts JW, Bierlaagh MC, Kroes S, Nieuwenhuijze NDA, Sonneveld van Kooten HN, Niemöller PJ, Verburg TF, Janssens HM, Muilwijk D, van Beuningen SFB, van der Ent CK, Beekman JM (2023) CFTR function restoration upon Elexacaftor/Tezacaftor/Ivacaftor treatment in patient-derived intestinal organoids with rare *CFTR* genotypes. Int J Mol Sci 24:14539. 10.3390/ijms241914539 37833986 10.3390/ijms241914539 PMC10572896

[219] Kleinfelder K, Villella VR, Hristodor AM, Laudanna C, Castaldo G, Amato F, Melotti P, Sorio C (2023) Theratyping of the rare CFTR genotype A559T in rectal organoids and nasal cells reveals a relevant response to Elexacaftor (VX-445) and Tezacaftor (VX-661) combination. Int J Mol Sci 24:10358. 10.3390/ijms241210358 37373505 10.3390/ijms241210358 PMC10299407

[220] Kondratyeva E, Bulatenko N, Melyanovskaya Y, Efremova A, Zhekaite E, Sherman V, Voronkova A, Asherova I, Polyakov A, Adyan T, Kovalskaia V, Bukharova T, Goldshtein D, Kutsev S (2022) Personalized selection of a CFTR modulator for a patient with a complex allele [L467F;F508del]. Curr Issues Mol Biol 44:5126–5138. 10.3390/cimb44100349 36286063 10.3390/cimb44100349 PMC9600521

[221] Ensinck MM, De Keersmaecker L, Ramalho AS, Cuyx S, Van Biervliet S, Dupont L, Christ F, Debyser Z, Vermeulen F, Carlon MS (2022) Novel CFTR modulator combinations maximise rescue of G85E and N1303K in rectal organoids. ERJ Open Res 8:00716–02021. 10.1183/23120541.00716-2021 35449760 10.1183/23120541.00716-2021 PMC9016267

[222] Kleinfelder K, Lotti V, Eramo A, Amato F, Lo Cicero S, Castelli G, Spadaro F, Farinazzo A, Dell'Orco D, Preato S, Conti J, Rodella L, Tomba F, Cerofolini A, Baldisseri E, Bertini M, Volpi S, Villella VR, Esposito S, Zollo I, Castaldo G, Laudanna C, Sorsher EJ, Hong J, Joshi D, Cutting G, Lucarelli M, Melotti P, Sorio C (2023) *In silico* analysis and theratyping of an ultra-rare CFTR genotype (W57G/A234D) in primary human rectal and nasal epithelial cells. iScience 26: 108180. 10.1016/j.isci.2023.108180. 10.1016/j.isci.2023.108180 PMC10660498 38026150

[223] Sadras I, Kerem E, Livnat G, Sarouk I, Breuer O, Reiter J, Gileles-Hillel A, Inbar O, Cohen M, Gamliel A, Stanleigh N, Gunawardena T, Bartlett C, Gonska T, Moraes T, Eckford PDW, Bear CE, Ratjen F, Kerem B, Wilschanski M, Shteinberg M, Cohen-Cymberknoh M (2023) Clinical and functional efficacy of elexacaftor/tezacaftor/ivacaftor in people with cystic fibrosis carrying the N1303K mutation. J Cyst Fibros 22:1062–1069. 10.1016/j.jcf.2023.06.001 37331863 10.1016/j.jcf.2023.06.001

[224] Efremova A, Kashirskaya N, Krasovskiy S, Melyanovskaya Y, Krasnova M, Mokrousova D, Bulatenko N, Kondratyeva E, Makhnach O, Bukharova T, Zinchenko R, Kutsev S, Goldshtein D (2024) Comprehensive assessment of CFTR modulators’ therapeutic efficiency for N1303K variant. Int J Mol Sci 25:2770. 10.3390/ijms25052770 38474016 10.3390/ijms25052770 PMC10931982

[225] Kleinfelder K, Melotti P, Hristodor AM, Fevola C, Taccetti G, Terlizzi V, Sorio C (2024) CFTR modulators response of S737F and T465N CFTR variants on patient-derived rectal organoids. Orphanet J Rare Dis 19:343. 10.1186/s13023-024-03334-3 39272186 10.1186/s13023-024-03334-3 PMC11401437

[226] Fainardi V, Cresta F, Sorio C, Melotti P, Pesce E, Deolmi M, Longo F, Karina K, Esposito S, Pisi G (2024) Elexacaftor/tezacaftor/ivacaftor in people with cystic fibrosis and rare mutations. Pediatr Pulmonol 59:3383–3390. 10.1002/ppul.27211 39212240 10.1002/ppul.27211

[227] Lo Cicero S, Castelli G, Blaconà G, Bruno SM, Sette G, Pigliucci R, Villella VR, Esposito S, Zollo I, Spadaro F, Maria R, Biffoni M, Cimino G, Amato F, Lucarelli M, Eramo A (2023) L1077P CFTR pathogenic variant function rescue by Elexacaftor-Tezacaftor-Ivacaftor in cystic fibrosis patient-derived air-liquid interface (ALI) cultures and organoids: in vitro guided personalized therapy of non-F508del patients. Respir Res 24:217. 10.1186/s12931-023-02516-0 37674160 10.1186/s12931-023-02516-0 PMC10483775

[228] Tomati V, Costa S, Capurro V, Pesce E, Pastorino C, Lena M, Sondo E, Di Duca M, Cresta F, Cristadoro S, Zara F, Galietta LJV, Bocciardi R, Castellani C, Lucanto MC, Pedemonte N (2023) Rescue by elexacaftor-tezacaftor-ivacaftor of the G1244E cystic fibrosis mutation’s stability and gating defects are dependent on cell background. J Cyst Fibros 22:525–537. 10.1016/j.jcf.2022.12.005 36543707 10.1016/j.jcf.2022.12.005

[229] Sondo E, Cresta F, Pastorino C, Tomati V, Capurro V, Pesce E, Lena M, Iacomino M, Baffico AM, Coviello D, Bandiera T, Zara F, Galietta LJV, Bocciardi R, Castellani C, Pedemonte N (2022) The L467F–F508del complex allele hampers pharmacological rescue of mutant CFTR by Elexacaftor/Tezacaftor/Ivacaftor in Cystic Fibrosis patients: The value of the ex vivo nasal epithelial model to address non-responders to CFTR-modulating drugs. Int J Mol Sci 23:3175. 10.3390/ijms23063175 35328596 10.3390/ijms23063175 PMC8952007

[230] Pallenberg ST, Held I, Dopfer C, Minso R, Nietert MM, Hansen G, Tümmler B, Dittrich AM (2023) Differential effects of ELX/TEZ/IVA on organ-specific CFTR function in two patients with the rare CFTR splice mutations c.273+1G>A and c.165–2A>G. Front Pharmacol 14:1153656. 10.3389/fphar.2023.1153656. 10.3389/fphar.2023.1153656 PMC10083416 37050906

[231] Liu Q, Sabirzhanova I, Yanda MK, Bergbower EAS, Boinot C, Guggino WB, Cebotaru L (2018) Rescue of CFTR NBD2 mutants N1303K and S1235R is influenced by the functioning of the autophagosome. J Cyst Fibros 17:582–594. 10.1016/j.jcf.2018.05.016 29936070 10.1016/j.jcf.2018.05.016 PMC6435267

[232] DeStefano S, Gees M, Hwang TC (2018) Physiological and pharmacological characterization of the N1303K mutant CFTR. J Cyst Fibros 17:573–581. 10.1016/j.jcf.2018.05.011 29887518 10.1016/j.jcf.2018.05.011 PMC7008954

[233] Vernon RM, Chong PA, Lin H, Yang Z, Zhou Q, Aleksandrov AA, Dawson JE, Riordan JR, Brouillette CG, Thibodeau PH, Forman-Kay JD (2017) Stabilization of a nucleotide-binding domain of the cystic fibrosis transmembrane conductance regulator yields insight into disease-causing mutations. J Biol Chem 292:14147–14164. 10.1074/jbc.M116.772335 28655774 10.1074/jbc.M116.772335 PMC5572908

[234] Dekkers JF, Gogorza Gondra RA, Kruisselbrink E, Vonk AM, Janssens HM, de Winter-de Groot KM, van der Ent CK, Beekman JM (2016) Optimal correction of distinct CFTR folding mutants in rectal cystic fibrosis organoids. Eur Respir J 48:451–458. 10.1183/13993003.01192-2015 27103391 10.1183/13993003.01192-2015

[235] Rapino D, Sabirzhanova I, Lopes-Pacheco M, Grover R, Guggino WB, Cebotaru L (2015) Rescue of NBD2 mutants N1303K and S1235R of CFTR by small-molecule correctors and transcomplementation. PLoS ONE 10:e0119796. 10.1371/journal.pone.0119796 25799511 10.1371/journal.pone.0119796 PMC4370480

[236] He L, Kennedy AS, Houck S, Aleksandrov A, Quinney NL, Cyr-Scully A, Cholon DM, Gentzsch M, Randell SH, Ren HY, Cyr DM (2021) DNAJB12 and Hsp70 triage arrested intermediates of N1303K-CFTR for endoplasmic reticulum-associated autophagy. Mol Biol Cell 32:538–553. 10.1091/mbc.E20-11-0688 33534640 10.1091/mbc.E20-11-0688 PMC8101465

[237] Huang Y, Paul G, Lee J, Yarlagadda S, McCoy K, Naren AP (2021) Elexacaftor/Tezacaftor/Ivacaftor improved clinical outcomes in a patient with N1303K-CFTR based on *in vitro* experimental evidence. Am J Respir Crit Care Med 204:1231–1235. 10.1164/rccm.202101-0090LE 34379998 10.1164/rccm.202101-0090LE PMC8759307

[238] Solomon GM, Linnemann RW, Rich R, Streby A, Buehler B, Hunter E, Vijaykumar K, Hunt WR, Brewington JJ, Rab A, Bai SP, Westbrook AL, McNicholas-Bevensee C, Hong J, Manfredi C, Barilla C, Suzuki S, Davis BR, Sorscher EJ (2024) Evaluation of elexacaftor-tezacaftor-ivacaftor treatment in individuals with cystic fibrosis and CFTR^N1303K^ in the USA: a prospective, multicentre, open-label, single-arm trial. Lancet Respir Med 12:947–957. 10.1016/S2213-2600(24)00205-4 39208836 10.1016/S2213-2600(24)00205-4

[239] Gentzsch M, Baker B, Cholon DM, Kam CW, McKinzie CJ, Despotes KA, Boyles SE, Quinney NL, Esther CR Jr, Ribeiro CMP (2024) Cystic fibrosis airway inflammation enables elexacaftor/tezacaftor/ivacaftor-mediated rescue of N1303K *CFTR* mutation. ERJ Open Res 10:00746–02023. 10.1183/23120541.00746-2023 38226069 10.1183/23120541.00746-2023 PMC10789252

[240] Rehman T, Pezzulo AA, Thurman AL, Zemans RL, Welsh MJ (2024) Epithelial responses to CFTR modulators are improved by inflammatory cytokines and impaired by antiinflammatory drugs. JCI Insight 9:e181836. 10.1172/jci.insight.181836 38888974 10.1172/jci.insight.181836 PMC11383177

[241] Ciobanu DZ, Liessi N, Tomati V, Capurro V, Bertozzi SM, Summa M, Bertorelli R, Loberto N, Dobi D, Aureli M, Nobbio L, Bandiera T, Pedemonte N, Bassi R, Armirotti A (2024) Tezacaftor is a direct inhibitor of sphingolipid delta-4 desaturase enzyme (DEGS). J Cyst Fibros 23:1167–1172. 10.1016/j.jcf.2024.05.004 38789319 10.1016/j.jcf.2024.05.004

[242] Liessi N, Tomati V, Capurro V, Loberto N, Garcia-Aloy M, Franceschi P, Aureli M, Pedemonte N, Armirotti A (2023) The combination elexacaftor/tezacaftor/ivacaftor (ETI) modulates the de novo synthethic pathway of ceramides in a genotype-independent manner. J Cyst Fibros 22:680–682. 10.1016/j.jcf.2023.04.012 37088636 10.1016/j.jcf.2023.04.012

[243] Teichgräber V, Ulrich M, Endlich N, Riethmüller J, Wilker B, De Oliveira-Munding CC, van Heeckeren AM, Barr ML, von Kürthy G, Schmid KW, Weller M, Tümmler B, Lang F, Grassme H, Döring G, Gulbins E (2008) Ceramide accumulation mediates inflammation, cell death and infection susceptibility in cystic fibrosis. Nat Med 14:382–391. 10.1038/nm1748 18376404 10.1038/nm1748

[244] Becker KA, Riethmüller J, Seitz AP, Gardner A, Boudreau R, Kamler M, Kleuser B, Schuchman E, Caldwell CC, Edwards MJ, Grassmé H, Brodlie M, Gulbins E (2018) Sphingolipids as targets for inhalation treatment of cystic fibrosis. Adv Drug Deliv Rev 133:66–75. 10.1016/j.addr.2018.04.015 29698625 10.1016/j.addr.2018.04.015

[245] Gardner AI, Wu Y, Verhaegh R, Liu Y, Wilker B, Soddemann M, Keitsch S, Edwards MJ, Haq IJ, Kamler M, Becker KA, Brodlie M, Gulbins E (2021) Interferon regulatory factor 8 regulates expression of acid ceramidase and infection susceptibility in cystic fibrosis. J Biol Chem 296:100650. 10.1016/j.jbc.2021.100650 33839155 10.1016/j.jbc.2021.100650 PMC8113888

[246] Westhölter D, Schumacher F, Wülfinghoff N, Sutharsan S, Strassburg S, Kleuser B, Horn PA, Reuter S, Gulbins E, Taube C, Welsner M (2022) CFTR modulator therapy alters plasma sphingolipid profiles in people with cystic fibrosis. J Cyst Fibros 21:713–720. 10.1016/j.jcf.2022.02.005 35168870 10.1016/j.jcf.2022.02.005

[247] Tümmler B (ed) (2022) Mutation-specific therapies in cystic fibrosis. UNI-MED, Bremen.

[248] Lévêque M, Mirval S, Barrault C, Fixe I, Coraux C, Sage E, Becq F, Vandebrouck C (2024) The F508del-CFTR trafficking correctors elexacaftor and tezacaftor are CFTR-independent Ca^2+^-mobilizing agonists normalizing abnormal Ca^2+^ levels in human airway epithelial cells. Respir Res 25:436. 10.1186/s12931-024-03059-8 39702307 10.1186/s12931-024-03059-8 PMC11660580

[249] Hampton TH, Barnaby R, Roche C, Nymon A, Fukutani KF, MacKenzie TA, Charpentier LA, Stanton BA (2024) Gene expression responses of CF airway epithelial cells exposed to elexacaftor/tezacaftor/ivacaftor suggest benefits beyond improved CFTR channel function. Am J Physiol Lung Cell Mol Physiol 327:L905–L916. 10.1152/ajplung.00272.2024 39437760 10.1152/ajplung.00272.2024 PMC11684945

[250] Burgel PR, Sermet-Gaudelus I, Durieu I, Kanaan R, Macey J, Grenet D, Porzio M, Coolen-Allou N, Chiron R, Marguet C, Douvry B, Dufeu N, Danner-Boucher I, Foucaud P, Lemonnier L, Girodon E, Da Silva J, Martin C, Reference FCF, Network study group, (2023) The French Compassionate Program of elexacaftor-tezacaftor-ivacaftor in people with cystic fibrosis with advanced lung disease and no F508del CFTR variant. Eur Respir J 16:2202437. 10.1183/13993003.02437-2022

[251] Burgel PR, Sermet-Gaudelus I, Girodon E, Durieu I, Houdouin V, Audousset C, Macey J, Grenet D, Porzio M, Murris-Espin M, Reix P, Baravalle M, Belleguic C, Mely L, Verhille J, Weiss L, Reynaud-Gaubert M, Mittaine M, Hamidfar R, Ramel S, Cosson L, Douvry B, Danner-Boucher I, Foucaud P, Roy C, Burnet E, Raynal C, Audrezet MP, Da Silva J, Martin C, Network FCFR, study group, (2024) The expanded French compassionate programme for elexacaftor-tezacaftor-ivacaftor use in people with cystic fibrosis without a F508del CFTR variant: a real-world study. Lancet Respir Med 12:888–900. 10.1016/S2213-2600(24)00208-X 39151434 10.1016/S2213-2600(24)00208-X

[252] Atteih SE, Armbruster CR, Hilliam Y, Rapsinski GJ, Bhusal JK, Krainz LL, Gaston JR, DuPont M, Zemke AC, Alcorn JF, Moore JA, Cooper VS, Lee SE, Forno E, Bomberger JM (2024) Effects of highly effective modulator therapy on the dynamics of the respiratory mucosal environment and inflammatory response in cystic fibrosis. Pediatr Pulmonol 59:1266–1273. 10.1002/ppul.26898 38353361 10.1002/ppul.26898 PMC11058019

[253] Westhölter D, Pipping J, Raspe J, Schmitz M, Sutharsan S, Straßburg S, Welsner M, Taube C, Reuter S (2023) Plasma levels of chemokines decrease during lexacaftor/tezacaftor/ivacaftor therapy in adults with cystic fibrosis. Heliyon 10:e23428. 10.1016/j.heliyon.2023.e23428 38173511 10.1016/j.heliyon.2023.e23428 PMC10761561

[254] Schaupp L, Addante A, Völler M, Fentker K, Kuppe A, Bardua M, Duerr J, Piehler L, Röhmel J, Thee S, Kirchner M, Ziehm M, Lauster D, Haag R, Gradzielski M, Stahl M, Mertins P, Boutin S, Graeber SY, Mall MA (2023) Longitudinal effects of elexacaftor/tezacaftor/ivacaftor on sputum viscoelastic properties, airway infection and inflammation in patients with cystic fibrosis. Eur Respir J 62:2202153. 10.1183/13993003.02153-2022 37414422 10.1183/13993003.02153-2022

[255] De Vuyst RC, Bennard E, Kam CW, McKinzie CJ, Esther CR (2023) Elexacaftor/tezacaftor/ivacaftor treatment reduces airway inflammation in cystic fibrosis. Pediatr Pulmonol 58:1592–1594. 10.1002/ppul.26334 36718851 10.1002/ppul.26334

[256] Sheikh S, Britt RD Jr, Ryan-Wenger NA, Khan AQ, Lewis BW, Gushue C, Ozuna H, Jaganathan D, McCoy K, Kopp BT (2023) Impact of elexacaftor-tezacaftor-ivacaftor on bacterial colonization and inflammatory responses in cystic fibrosis. Pediatr Pulmonol 58:825–833. 10.1002/ppul.26261 36444736 10.1002/ppul.26261 PMC9957929

[257] Caley LR, Gillgrass L, Zagoya C, Saumtally H, Duckstein F, White H, Mainz JG, Peckham DG (2025) Longer term follow-up of abdominal symptoms (CFAbd-Score) after initiation of Elexacaftor / Tezacaftor / Ivacaftor in adults with cystic fibrosis. J Cyst Fibros S1569–1993(25):00010–00014. 10.1016/j.jcf.2025.01.010 10.1016/j.jcf.2025.01.010 39814671

[258] Zampoli M, Morrow BM, Paul G (2023) Real-world disparities and ethical considerations with access to CFTR modulator drugs: Mind the gap! Front Pharmacol 14:1163391. 10.3389/fphar.2023.1163391 37050905 10.3389/fphar.2023.1163391 PMC10083423

[259] Kerem E (2025) Expanding the impact of new Cystic Fibrosis therapies in low- and middle-income countries. Pediatr Pulmonol 60(Suppl 1):S90–S91. 10.1002/ppul.27362 39625248 10.1002/ppul.27362 PMC11921072

[260] Vonk SEM, Lub R, Weersink EJM, Beuers U, Mathôt RAA, Kemper EM, Altenburg J; Amsterdam Mucociliary Clearance Disease Research Group (2024) Stepwise introduction of Elexacaftor-Tezacaftor-Ivacaftor in patients with Cystic Fibrosis and liver cirrhosis Child-Pugh A or B using clinical and therapeutic drug monitoring: A case series. Clin Ther 46:154-158. 10.1016/j.clinthera.2023.11.003 10.1016/j.clinthera.2023.11.003 38042631

[261] Hong E, Li R, Shi A, Almond LM, Wang J, Khudari AZ, Haddad S, Sislyan S, Angelich M, Chung PS, Rao AP, Beringer PM (2023) Safety of elexacaftor/tezacaftor/ivacaftor dose reduction: Mechanistic exploration through physiologically based pharmacokinetic modeling and a clinical case series. Pharmacotherapy 43:291–299. 10.1002/phar.2786 36866442 10.1002/phar.2786

[262] Hong E, Almond LM, Chung PS, Rao AP, Beringer PM (2022) Physiologically based pharmacokinetic modeling to guide management of drug interactions between Elexacaftor-Tezacaftor-Ivacaftor and antibiotics for the treatment of nontuberculous mycobacteria. Antimicrob Agents Chemother 66:e0110422. 10.1128/aac.01104-22 36286508 10.1128/aac.01104-22 PMC9664863

[263] Somerville L, Borish L, Noth I, Albon D (2024) Modulator-refractory cystic fibrosis: Defining the scope and challenges of an emerging at-risk population. Ther Adv Respir Dis 18:17534666241297876. 10.1177/17534666241297877 39543951 10.1177/17534666241297877 PMC11565698

[264] Georgiopoulos AM, Tillman EM (2025) The impact of CFTR modulators on mental health: Moving the field forward. J Cyst Fibros 24:5–7. 10.1016/j.jcf.2024.12.002 39701903 10.1016/j.jcf.2024.12.002

[265] Bathgate CJ, Fedele DA, Tillman EM, He J, Everhart RS, Reznikov LR, Liu FF, Kirby K, Raffensperger K, Traver K, Riekert KA, Powers SW, Georgiopoulos AM (2025) Elexacaftor/tezacaftor/ivacaftor and mental health: A workshop report from the Cystic Fibrosis Foundation’s Prioritizing Research in Mental Health working group. J Cyst Fibros 24:301–309. 10.1016/j.jcf.2024.11.006 39592379 10.1016/j.jcf.2024.11.006

[266] O’Connor J, Nazareth D, Wat D, Southern KW, Frost F (2025) Regulatory adverse drug reaction analyses support a temporal increase in psychiatric reactions after initiation of cystic fibrosis combination modulator therapies. J Cyst Fibros 24:30–32. 10.1016/j.jcf.2024.09.010 39299889 10.1016/j.jcf.2024.09.010

[267] Collins B, Fortner C, Cotey A, Esther CRJ, Trimble A (2022) Drug exposure to infants born to mothers taking Elexacaftor, Tezacaftor, and Ivacaftor. J Cyst Fibros 21:725–727. 10.1016/j.jcf.2021.12.004 34952795 10.1016/j.jcf.2021.12.004 PMC9213569

[268] Evans IA, Sun X, Liang B, Vegter AR, Guo L, Lynch TJ, Zhang Y, Zhang Y, Yi Y, Yang Y, Feng Z, Park SY, Shonka A, McCumber H, Qi L, Wu P, Liu G, Lacina A, Wang K, Gibson-Corley KN, Meyerholz DK, Limoli DH, Rosen BH, Yan Z, Bartels DJ, Engelhardt JF (2024) In utero and postnatal ivacaftor/lumacaftor therapy rescues multiorgan disease in CFTR-F508del ferrets. JCI Insight 9:e157229. 10.1172/jci.insight.157229 38646935 10.1172/jci.insight.157229 PMC11141870

[269] Metcalf A, Martiniano SL, Sagel SD, Zaretsky MV, Zemanick ET, Hoppe JE (2024) Outcomes of prenatal use of elexacaftor/tezacaftor/ivacaftor in carrier mothers to treat meconium ileus in fetuses with cystic fibrosis. J Cyst Fibros S1569–1993(24):01843–01845. 10.1016/j.jcf.2024.11.011 10.1016/j.jcf.2024.11.011 39645477

[270] Kowalik A, Roberts E, Harris AH, Sund M, Wird S, Kvist O, Hjelte L (2024) Clinical outcomes of two infants with cystic fibrosis, including presence of the vas deferens, born to a woman with cystic fibrosis taking CFTR modulators during both pregnancies. J Cyst Fibros 23:1027–1030. 10.1016/j.jcf.2024.06.003 38876833 10.1016/j.jcf.2024.06.003

[271] Jain R, Magaret A, Vu PT, VanDalfsen JM, Keller A, Wilson A, Putman MS, Mayer-Hamblett N, Esther CR Jr, Taylor-Cousar JL (2022) Prospectively evaluating maternal and fetal outcomes in the era of CFTR modulators: the MAYFLOWERS observational clinical trial study design. BMJ Open Respir Res 9:e001289. 10.1136/bmjresp-2022-001289 35710144 10.1136/bmjresp-2022-001289 PMC9204448

[272] Felton I, Downes A, Bokobza I, Weitnauer L, Davies JC (2024) “Shifting sands in cystic fibrosis”: impacts of CFTR modulators on reproductive health in people with cystic fibrosis and challenges related to *in utero* exposure. Expert Opin Pharmacother 25:2243–2252. 10.1080/14656566.2024.2426677 39543810 10.1080/14656566.2024.2426677

[273] Keating C, Yonker LM, Vermeulen F, Prais D, Linnemann RW, Trimble A, Kotsimbos T, Mermis J, Braun AT, O’Carroll M, Sutharsan S, Ramsey B, Mall MA, Taylor-Cousar JL, McKone EF, Tullis E, Floreth T, Michelson P, Sosnay PR, Nair N, Zahigian R, Martin H, Ahluwalia N, Lam A, Horsley A (2025) Vanzacaftor-tezacaftor-deutivacaftor versus elexacaftor-tezacaftor-ivacaftor in individuals with cystic fibrosis aged 12 years and older (SKYLINE Trials VX20-121-102 and VX20-121-103): results from two randomised, active-controlled, phase 3 trials. Lancet Respir Med 13:256–271. 10.1016/S2213-2600(24)00411-9 39756424 10.1016/S2213-2600(24)00411-9 PMC12184100

[274] Hoppe JE, Kasi AS, Pittman JE, Jensen R, Thia LP, Robinson P, Tirakitsoontorn P, Ramsey B, Mall MA, Taylor-Cousar JL, McKone EF, Tullis E, Salinas DB, Zhu J, Chen YC, Rodriguez-Romero V, Sosnay PR, Davies G (2025) Vanzacaftor-tezacaftor-deutivacaftor for children aged 6–11 years with cystic fibrosis (RIDGELINE Trial VX21-121-105): an analysis from a single-arm, phase 3 trial. Lancet Respir Med 13:244–255. 10.1016/S2213-2600(24)00407-7 39756425 10.1016/S2213-2600(24)00407-7 PMC12126198

[275] Sionna therapeutics (2024) Waltham. https://www.sionnatx.com/about-us/. Accessed 30 Dec 2024.

[276] Drug development pipeline (2024) Cystic Fibrosis Foundation, Bethesda. https://apps.cff.org/trials/pipeline/. Accessed 30 Dec 2024.

[277] Oliver KE, Carlon MS, Pedemonte N, Lopes-Pacheco M (2023) The revolution of personalized pharmacotherapies for cystic fibrosis: what does the future hold? Expert Opin Pharmacother 24:1545–1565. 10.1080/14656566.2023.2230129 37379072 10.1080/14656566.2023.2230129 PMC10528905

[278] Pinto MC, Botelho HM, Silva IAL, Railean V, Neumann B, Pepperkok R, Schreiber R, Kunzelmann K, Amaral MD (2022) Systems Approaches to Unravel Molecular Function: High-content siRNA Screen Identifies TMEM16A Traffic Regulators as Potential Drug Targets for Cystic Fibrosis. J Mol Biol 434:167436. 10.1016/j.jmb.2021.167436 34990652 10.1016/j.jmb.2021.167436

[279] Balázs A, Mall MA (2018) Role of the SLC26A9 chloride channel as disease modifier and potential therapeutic target in Cystic Fibrosis. Front Pharmacol 9:1112. 10.3389/fphar.2018.01112 30327603 10.3389/fphar.2018.01112 PMC6174851

[280] Pinto MC, Quaresma MC, Silva IAL, Railean V, Ramalho SS, Amaral MD (2021) Synergy in Cystic Fibrosis Therapies: Targeting SLC26A9. Int J Mol Sci 22:13064. 10.3390/ijms222313064 34884866 10.3390/ijms222313064 PMC8658147

[281] Kunzelmann K, Ousingsawat J, Cabrita I, Doušová T, Bähr A, Janda M, Schreiber R, Benedetto R (2019) TMEM16A in Cystic Fibrosis: Activating or inhibiting? Front Pharmacol 10:3. 10.3389/fphar.2019.00003 30761000 10.3389/fphar.2019.00003 PMC6362895

[282] Kunzelmann K, Centeio R, Ousingsawat J, Talbi K, Seidler U, Schreiber R (2023) SLC26A9 in airways and intestine: secretion or absorption? Channels (Austin) 17:2186434. 10.1080/19336950.2023.2186434 36866602 10.1080/19336950.2023.2186434 PMC9988340

[283] Salari A, Xiu R, Amiri M, Pallenberg ST, Schreiber R, Dittrich AM, Tümmler B, Kunzelmann K, Seidler U (2023) The anion channel TMEM16a/Ano1 modulates CFTR activity, but does not function as an apical anion channel in colonic epithelium from Cystic Fibrosis patients and healthy individuals. Int J Mol Sci 24:14214. 10.3390/ijms241814214 37762516 10.3390/ijms241814214 PMC10531629

[284] Allaire NE, Griesenbach U, Kerem B, Lueck JD, Stanleigh N, Oren YS (2023) Gene, RNA, and ASO-based therapeutic approaches in Cystic Fibrosis. J Cyst Fibros 22(Suppl 1):S39–S44. 10.1016/j.jcf.2022.12.016 36658041 10.1016/j.jcf.2022.12.016 PMC10012168

[285] Oren YS, Irony-Tur Sinai M, Golec A, Barchad-Avitzur O, Mutyam V, Li Y, Hong J, Ozeri-Galai E, Hatton A, Leibson C, Carmel L, Reiter J, Sorscher EJ, Wilton SD, Kerem E, Rowe SM, Sermet-Gaudelus I, Kerem B (2021) Antisense oligonucleotide-based drug development for Cystic Fibrosis patients carrying the 3849+10 kb C-to-T splicing mutation. J Cyst Fibros 20:865–875. 10.1016/j.jcf.2021.06.003 34226157 10.1016/j.jcf.2021.06.003 PMC8464507

[286] Oren YS, Avizur-Barchad O, Ozeri-Galai E, Elgrabli R, Schirelman MR, Blinder T, Stampfer CD, Ordan M, Laselva O, Cohen-Cymberknoh M, Kerem E, Bear CE, Kerem B (2022) Antisense oligonucleotide splicing modulation as a novel Cystic Fibrosis therapeutic approach for the W1282X nonsense mutation. J Cyst Fibros 21:630–636. 10.1016/j.jcf.2021.12.012 34972649 10.1016/j.jcf.2021.12.012

[287] Kim YJ, Sivetz N, Layne J, Voss DM, Yang L, Zhang Q, Krainer AR (2022) Exon-skipping antisense oligonucleotides for cystic fibrosis therapy. Proc Natl Acad Sci U S A 119:e2114858118. 10.1073/pnas.2114858118 35017301 10.1073/pnas.2114858118 PMC8784140

[288] Kim YJ, Nomakuchi T, Papaleonidopoulou F, Yang L, Zhang Q, Krainer AR (2022) Gene-specific nonsense-mediated mRNA decay targeting for cystic fibrosis therapy. Nat Commun 13:2978. 10.1038/s41467-022-30668-y 35624092 10.1038/s41467-022-30668-y PMC9142507

[289] Ozeri-Galai E, Friedman L, Barchad-Avitzur O, Markovetz MR, Boone W, Rouillard KR, Stampfer CD, Oren YS, Hill DB, Kerem B, Hart G (2023) Delivery characterization of SPL84 inhaled antisense oligonucleotide drug for 3849 + 10 kb C- > T Cystic Fibrosis patients. Nucleic Acid Ther 33:306–318. 10.1089/nat.2023.0015 37643307 10.1089/nat.2023.0015

[290] Caraco Y, Wanounou M, Blotnick S, Friedman L, Cohen A, Hart G, Kerem E (2024) A phase I study assessing the safety and tolerability of SPL84, an inhaled antisense oligonucleotide for treatment of cystic fibrosis patients with the 3849 +10kb C->T. J Cyst Fibros 2024 Nov 4: S1569-1993(24)01798-3. 10.1016/j.jcf.2024.10.004 10.1016/j.jcf.2024.10.004 39500647

[291] Lee RE, Lewis CA, He L, Bulik-Sullivan EC, Gallant SC, Mascenik TM, Dang H, Cholon DM, Gentzsch M, Morton LC, Minges JT, Theile JW, Castle NA, Knowles MR, Kimple AJ, Randell SH (2022) Small-molecule eRF3a degraders rescue CFTR nonsense mutations by promoting premature termination codon readthrough. J Clin Invest 132:e154571. 10.1172/JCI154571 35900863 10.1172/JCI154571 PMC9479597

[292] Porter JJ, Ko W, Sorensen EG, Lueck JD (2024) Optimization of ACE-tRNAs function in translation for suppression of nonsense mutations. Nucleic Acids Res 52:14112–14132. 10.1093/nar/gkae1112 39673265 10.1093/nar/gkae1112 PMC11662937

[293] Albers S, Allen EC, Bharti N, Davyt M, Joshi D, Perez-Garcia CG, Santos L, Mukthavaram R, Delgado-Toscano MA, Molina B, Kuakini K, Alayyoubi M, Park KJ, Acharya G, Gonzalez JA, Sagi A, Birket SE, Tearney GJ, Rowe SM, Manfredi C, Hong JS, Tachikawa K, Karmali P, Matsuda D, Sorscher EJ, Chivukula P, Ignatova Z (2023) Engineered tRNAs suppress nonsense mutations in cells and in vivo. Nature 618:842–848. 10.1038/s41586-023-06133-1 37258671 10.1038/s41586-023-06133-1 PMC10284701

[294] Bharti N, Santos L, Davyt M, Behrmann S, Eichholtz M, Jimenez-Sanchez A, Hong JS, Rab A, Sorscher EJ, Albers S, Ignatova Z (2024) Translation velocity determines the efficacy of engineered suppressor tRNAs on pathogenic nonsense mutations. Nat Commun 15:2957. 10.1038/s41467-024-47258-9 38580646 10.1038/s41467-024-47258-9 PMC10997658

[295] Chen PJ, Liu DR (2023) Prime editing for precise and highly versatile genome manipulation. Nat Rev Genet 24:161–177. 10.1038/s41576-022-00541-1 36344749 10.1038/s41576-022-00541-1 PMC10989687

[296] Vaidyanathan S, Kerschner JL, Paranjapye A, Sinha V, Lin B, Bedrosian TA, Thrasher AJ, Turchiano G, Harris A, Porteus MH (2024) Investigating adverse genomic and regulatory changes caused by replacement of the full-length *CFTR* cDNA using Cas9 and AAV. Mol Ther Nucleic Acids 35:102134. 10.1016/j.omtn.2024.102134 38384445 10.1016/j.omtn.2024.102134 PMC10879780

[297] Kanke KL, Rayner RE, Bozik J, Abel E, Venugopalan A, Suu M, Nouri R, Stack JT, Guo G, Vetter TA, Cormet-Boyaka E, Hester ME, Vaidyanathan S (2024) Single-stranded DNA with internal base modifications mediates highly efficient knock-in in primary cells using CRISPR-Cas9. Nucleic Acids Res 52:13561–13576. 10.1093/nar/gkae1069 39569586 10.1093/nar/gkae1069 PMC11662658

[298] Wei T, Sun Y, Cheng Q, Chatterjee S, Traylor Z, Johnson LT, Coquelin ML, Wang J, Torres MJ, Lian X, Wang X, Xiao Y, Hodges CA, Siegwart DJ (2023) Lung SORT LNPs enable precise homology-directed repair mediated CRISPR/Cas genome correction in cystic fibrosis models. Nat Commun 14:7322. 10.1038/s41467-023-42948-2 37951948 10.1038/s41467-023-42948-2 PMC10640563

[299] Bulcaen M, Kortleven P, Liu RB, Maule G, Dreano E, Kelly M, Ensinck MM, Thierie S, Smits M, Ciciani M, Hatton A, Chevalier B, Ramalho AS, Solvas CI, X, Debyser Z, Vermeulen F, Gijsbers R, Sermet-Gaudelus I, Cereseto A, Carlon MS, (2024) Prime editing functionally corrects cystic fibrosis-causing CFTR mutations in human organoids and airway epithelial cells. Cell Rep Med 5:101544. 10.1016/j.xcrm.2024.101544 38697102 10.1016/j.xcrm.2024.101544 PMC11148721

[300] Sousa AA, Hemez C, Lei L, Traore S, Kulhankova K, Newby GA, Doman JL, Oye K, Pandey S, Karp PH, McCray PB Jr, Liu DR (2025) Systematic optimization of prime editing for the efficient functional correction of CFTR F508del in human airway epithelial cells. Nat Biomed Eng 9:7–21. 10.1038/s41551-024-01233-3 38987629 10.1038/s41551-024-01233-3 PMC11754097

[301] Witten J, Raji I, Manan RS, Beyer E, Bartlett S, Tang Y, Ebadi M, Lei J, Nguyen D, Oladimeji F, Jiang AY, MacDonald E, Hu Y, Mughal H, Self A, Collins E, Yan Z, Engelhardt JF, Langer R, Anderson DG (2024) Artificial intelligence-guided design of lipid nanoparticles for pulmonary gene therapy. Nat Biotechnol. 10.1038/s41587-024-02490-y 39658727 10.1038/s41587-024-02490-y PMC12149338

[302] Soto MR, Lewis MM, Leal J, Pan Y, Mohanty RP, Veyssi A, Maier EY, Heiser BJ, Ghosh D (2024) Discovery of peptides for ligand-mediated delivery of mRNA lipid nanoparticles to cystic fibrosis lung epithelia. Mol Ther Nucleic Acids 35:102375. 10.1016/j.omtn.2024.102375 39640013 10.1016/j.omtn.2024.102375 PMC11617931

[303] Moiseenko A, Sinadinos A, Sergijenko A, Pineault K, Saleh A, Nekola K, Strang N, Eleftheraki A, Boyd AC, Davies JC, Gill DR, Hyde SC, McLachlan G, Rath T, Rothe M, Schambach A, Hobbie S, Schuler M, Maier U, Thomas MJ, Mennerich D, Schmidt M, Griesenbach U, Alton EWFW, Kreuz S (2024) Pharmacological and pre-clinical safety profile of rSIV.F/HN, a hybrid lentiviral vector for cystic fibrosis gene therapy. Eur Respir J 21:2301683. 10.1183/13993003.01683-2023. 10.1183/13993003.01683-2023 PMC11780724 39174284

[304] Davies JC, Polineni D, Boyd AC, Donaldson S, Gill DR, Griesenbach U, Hyde SC, Jain R, McLachlan G, Mall MA, Alton EWFW (2024) Lentiviral gene therapy for Cystic Fibrosis: A promising approach and first-in-human trial. Am J Respir Crit Care Med 210:1398–1408. 10.1164/rccm.202402-0389CI 39236265 10.1164/rccm.202402-0389CI PMC11716034

[305] Excoffon KJDA, Lin S, Narayan PKL, Sitaraman S, Jimah AM, Fallon TT, James ML, Glatfelter MR, Limberis MP, Smith MD, Guffanti G, Kolbeck R (2024) SP-101, A novel adeno-associated virus gene therapy for the treatment of Cystic Fibrosis, mediates functional correction of primary human airway epithelia from donors with Cystic Fibrosis. Hum Gene Ther 35:695–709. 10.1089/hum.2024.063 39155805 10.1089/hum.2024.063

[306] Flotte TR (2024) The road less traveled: Slow but steady progress toward Cystic Fibrosis Gene Therapy by the UK Respiratory Gene Therapy Consortium. Am J Respir Crit Care Med 210:1387–1389. 10.1164/rccm.202408-1516ED 39265185 10.1164/rccm.202408-1516ED PMC11716029

[307] Davies JC, Mall MA, Polineni D, Donaldson SH, Fajac I, Jain R, Rubin BK, Boyd AC, Gill DR, Griesenbach U, Hyde SC, McLachlan G, Avis M, Diefenbach C, Sigmund R, Seibold W, Gupta A, Alton E (2024) Lenticlair™ 1: A phase 1/2 trial evaluating the safety, tolerability and efficacy of an inhaled F/HN-pseudotyped lentiviral vector for CF gene therapy in people for CF ineligible for CFTR modulators. J Cyst Fibros 23(Suppl 1):S80

[308] Marsh R, Santos CD, Yule A, Dellschaft NS, Hoad CL, Ng C, Major G, Smyth AR, Rivett D, van der Gast C (2024) Impact of extended Elexacaftor/Tezacaftor/Ivacaftor therapy on the gut microbiome in cystic fibrosis. J Cyst Fibros 23:967–976. 10.1016/j.jcf.2024.05.002 38749891 10.1016/j.jcf.2024.05.002

